# Combining Ketamine and Internet-Based Cognitive Behavioral Therapy for the Treatment of Posttraumatic Stress Disorder: Protocol for a Randomized Controlled Trial

**DOI:** 10.2196/30334

**Published:** 2021-07-20

**Authors:** Aaron Emile Philipp-Muller, Taras Reshetukha, Gustavo Vazquez, Roumen Milev, Dawn Armstrong, Jasleen Jagayat, Nazanin Alavi

**Affiliations:** 1 Centre for Neuroscience Studies Queen's University Kingston, ON Canada; 2 Department of Psychiatry Queen's University Kingston, ON Canada

**Keywords:** mental health, PTSD, psychotherapy, cognitive behavioral therapy, online, internet, electronic, virtual, mental health care, ketamine

## Abstract

**Background:**

Over one third of patients with posttraumatic stress disorder (PTSD) do not respond to current interventions. Ketamine presents a potential treatment option; however, its effects are temporary. Administering ketamine alongside psychotherapy is one potential means of prolonging its effects; however, only a few studies have investigated this treatment method to date, and none have tested ketamine with internet-based or electronically delivered cognitive behavioral therapy (e-CBT).

**Objective:**

This open-label randomized controlled trial aims to assess the efficacy of a combined treatment method of subanesthetic intravenous ketamine and e-CBT for treating patients with PTSD.

**Methods:**

In total, 20 patients with refractory PTSD recruited from a community clinic will be randomly assigned to either an experimental group (n=10), receiving a combination of ketamine and therapist-administered e-CBT over 14 weeks, or a waitlist control group (n=10), receiving the experimental treatment after 14 weeks. Both groups will be assessed for the symptoms of PTSD and comorbid disorders before treatment, at two midway points, and at the end of the experiment.

**Results:**

PTSD symptoms of participants in the experimental group are expected to improve significantly more than those of participants in the waitlist control group (*P*=.05) with a large effect size (*η^2^*=0.14).

**Conclusions:**

This is the first study to assess the relationship between e-CBT and ketamine and their combined ability to treat refractory PTSD. If successful, this study will open web-based, asynchronous therapeutic options for patients with PTSD and will provide new insights into the functional role of glutamate in trauma-related disorders as well as in learning, memory, and fear extinction.

**Trial Registration:**

ClinicalTrials.gov NCT04771767; https://clinicaltrials.gov/ct2/show/NCT04771767.

**International Registered Report Identifier (IRRID):**

PRR1-10.2196/30334

## Introduction

### Challenges in Treating Posttraumatic Stress Disorder

Posttraumatic stress disorder (PTSD) is a chronic and debilitating mental illness that affects 3.5% of North American adults, or approximately 28 million people, with a lifetime prevalence of 8% [1]. PTSD develops after direct or indirect exposure to a psychologically traumatic incident, thereby leading to a host of cognitive, emotional, and behavioral symptoms [1]. Although most patients recover after a psychological trauma, a considerable minority of individuals remain chronically symptomatic or experience a delayed onset of PTSD [2]. PTSD is a particularly refractory disorder that persists for years, with many patients still displaying symptoms 20 years after a trauma, with prevalence often increasing over time after a trauma [3,4]. PTSD is also associated with high comorbidity rates with depression, anxiety, and substance abuse disorders [5,6]. Furthermore, patients with PTSD are 4 times more likely to attempt suicide than trauma survivors without PTSD [7]. Taken together, these features highlight the urgent need of effective treatments for this disorder.

A number of empirically supported psychotherapeutic treatments are available for PTSD, with trauma-focused cognitive behavioral therapy (TF-CBT) and eye movement desensitization and reprocessing therapy being the most effective [8,9]. However, psychotherapeutic treatments have a considerable nonresponse rate [10,11]. A number of pharmacotherapies have also become available for treating PTSD, with selective serotonin reuptake inhibitors such as paroxetine and sertraline showing the greatest success [12]. Unfortunately, pharmacotherapies have a larger nonresponse rate than psychotherapeutic treatments [12]. As a result, a sizeable proportion of patients with PTSD remain resistant to treatment. The objective of this study is to provide a greater reduction in symptoms with a combination treatment of ketamine and electronically delivered cognitive behavioral therapy (e-CBT) as compared with currently available therapeutic options for treatment-resistant patients.

### Ketamine and PTSD

Ketamine offers a promising research avenue for treating refractory PTSD. It is primarily a glutamate antagonist at the N-methyl-D-aspartate (NMDA) receptor and has achieved considerable success in rapidly reducing symptoms of mood disorders [13]. Ketamine is believed to function by disengaging an established pattern of thought [14,15], which in the case of PTSD would involve counteracting the impaired fear extinction seen in PTSD as ketamine increases neuroplasticity toward fear learning [16,17]. It is important to note that the exact mechanism of action in the treatment of emotional disorders is only partly understood. In terms of the development of PTSD, recent advances have generally implicated the NMDA receptor, where rodents subjected to chronic stress have elevated gene expression for producing NMDA receptors in the ventral hippocampus in comparison with control subjects [18]. Moreover, in humans, the prefrontal cortex and amygdala are connected by glutamatergic projections, suggesting that glutamate mediates a fear response [19]. Ketamine, in turn, has achieved considerable success in treating refractory PTSD, where it reduces symptoms significantly more than an active placebo for treatment-resistant patients [20].

Although ketamine opens treatment options to a new patient cohort, a purely pharmacological approach would be an oversimplification given the nature of PTSD, as the disorder develops in the wake of a trauma and cannot develop from pathological neurochemistry or neuroanatomy alone. Moreover, ketamine’s effects wear off in less than a week [20,21] and repeated infusions can induce potentially negative long-term outcomes on cognitive and physical health [22]. One potential way to prolong its effects is to capitalize on ketamine’s role in facilitating fear extinction by combining it with psychotherapy. This is partly attributed to psychotherapeutic interventions having greater longevity than pharmacological techniques for reducing PTSD symptoms [23]. Currently, there have been very few studies combining ketamine and psychotherapy to treat PTSD. As of May 2021, there were 4 studies listed on the ClinicalTrials.gov database that investigated ketamine used in combination with psychotherapy for treating PTSD (NCT02727998, NCT02766192, NCT04560660, and NCT03960658). These studies have shown promising results, where patients who were administered a combination of ketamine and prolonged exposure therapy or mindfulness-based extinction and reconsolidation therapy had a greatly prolonged therapeutic response as compared with ketamine alone [24,25]. The proposed study would therefore attempt to build on these findings by examining other types of psychotherapy in conjunction with ketamine for treating PTSD.

### This Study

In this study, we will focus on TF-CBT. As mentioned earlier, TF-CBT is a well-established form of psychotherapy and is considered among the most effective forms of psychotherapy for PTSD immediately after treatment and at follow-up [8,26,27]. However, as mentioned earlier, a considerable proportion of patients with PTSD still do not respond to TF-CBT. One challenge associated with cognitive behavioral therapy (CBT) is to optimize inhibitory learning, which is inherently challenging for patients with PTSD [28,29]. Ketamine treatment may address this challenge because it acts to boost neuroplasticity. Another challenge with CBT is its resource-intensive nature, with large associated costs and waiting times [30,31]. One variant of CBT that addresses this second challenge is e-CBT, which has equivalent efficacy as face-to-face CBT for treating PTSD as observed in several meta-analyses [32,33]. However, no studies have yet examined the interaction of ketamine treatment with e-CBT or CBT in general. Therefore, this study assessed whether ketamine combined with e-CBT significantly reduces the symptoms of PTSD in treatment-resistant patients. We hypothesize that ketamine treatment alongside e-CBT will reduce symptoms of treatment-resistant PTSD more significantly than a waitlist control, thereby providing preliminary evidence that these two treatments can be successfully combined.

## Methods

### Study Design Overview

**Figure 1:**
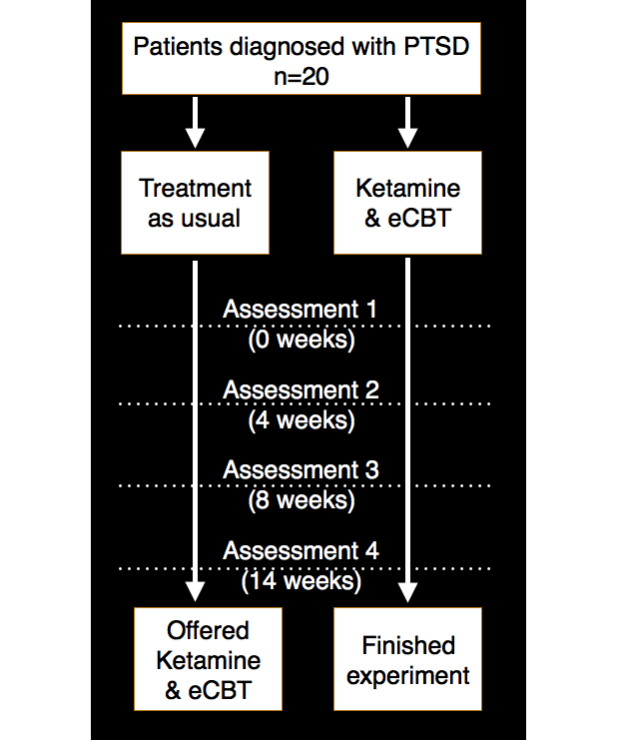
Summary of the experimental design and time course. This study will follow a 25- to 39-week period, including an 8-week period of stable treatment before recruitment, 3 weeks of screening, 14 weeks of experimental treatment or waitlist treatment, and 14 weeks of postexperiment treatment for patients in the waitlist condition. eCBT: electronically delivered cognitive behavioral therapy; PTSD: posttraumatic stress disorder.

This study had a randomized, open-label, parallel design. Participants in the intervention group will be offered a combined treatment involving a 14-week trauma-focused e-CBT program alongside 6 doses of intravenous (IV) ketamine. Participants in the control group will be placed on a 14-week waitlist receiving treatment as usual. Quantitative analyses will be conducted using standard measures of PTSD symptom severity and symptom severity of comorbid disorders. The Queen’s University Health Sciences and the Affiliated Teaching Hospitals Research Ethics Board approved this protocol. Figure 1 presents a summary of the experimental design.

### Participants

Participants (n=20) aged 18-65 years were enrolled in the study based on referrals from outpatient psychiatry at Hotel Dieu Hospital in Kingston, Ontario, Canada. A power analysis based on the most conservative effect size found in previous works [24,25] demonstrates that at least 5 participants will be required from each treatment group for a power of at least 0.9 (Cohen *d*=1.21; *P*=.05). In addition, although there has been a very strong effect observed in previous work, this study will take a conservative approach to the sample size, recruiting 10 participants per treatment group.

### Recruitment

The referring physicians will first be given flyers containing a brief description of the study with inclusion and exclusion criteria and contact information. They will then provide incoming patients that match the basic criteria with a copy of the flyer, informing them of this study. Patients who are interested in participating will contact the study coordinator, who will call the patient to conduct a prescreening interview and ask basic questions to determine if the patient may be eligible. If the patient passes the interview, the patient will proceed to the screening phase.

### Screening

The participant will participate in three separate screening appointments as follows:

#### First Screening Visit

At this 1-hour virtual meeting, participants will meet with one of the psychiatrists on the research team who will conduct an interview using the Clinician-Administered PTSD Scale (CAPS-5) [34], which is a detailed psychiatric interview that will be used to confirm the diagnosis of PTSD using the *Diagnostic and Statistical Manual of Mental Disorders, 5th edition* (*DSM-5*) and to determine the exact severity of each participant’s case. Participants will also be screened for study-specific inclusion and exclusion criteria. Patients who qualify for this study will satisfy all inclusion and exclusion criteria and will have a score of at least 50 on the CAPS-5 with the required distribution of symptoms across subcategories as outlined in the *DSM-5* to qualify at least as moderate presentation. Participants will also provide basic demographic data, including age and sex.

The inclusion criteria are as follows:

1. Provide oral consent.
2. Patients age 18-65 years at the start of the study.
3. Patients will be diagnosed with PTSD by a psychiatrist on the team as outlined in the *DSM-5* to qualify at least as a moderate presentation on the CAPS-5 with a score of at least 50.
4. Patients will be resistant to treatment, having previously received at least two different types of treatment, including any combination of selective serotonin reuptake inhibitors, serotonin norepinephrine reuptake inhibitors or TF-CBT, and all previous treatments will have produced less than a 50% reduction in the participant’s symptoms.
5. Patients will be on stable treatment for at least eight weeks before screening, with no alterations to the treatment regimen.
6. If a participant is female and of childbearing potential, then an effective method of contraception must be used as ketamine can be harmful to the neural development of an embryo or fetus.
7. Participants must be able to speak and read in English and have consistent and reliable access to the internet to complete the e-CBT course.
8. Participants must agree to adhere to the study protocol.

The exclusion criteria are as follows:

1. Previous hypersensitivity or allergy to ketamine
2. Hypomanic or manic episodes, bipolar disorder, acute psychosis, or schizophrenia
3. Opioid use disorder, current use of opioids, or treatment with naltrexone
4. Currently pregnant, postpartum, or breastfeeding
5. Untreated or inadequately controlled hypertension or cardiovascular disease
6. Elevated intracranial pressure
7. Renal or hepatic disease
8. Antisocial personality disorder or active homicidal ideation

#### Second Screening Visit

At this virtual meeting, participants will meet with a research assistant who will interview them using the Mini-International Neuropsychiatric Interview [35], designed to assess symptoms across a wide array of psychiatric conditions.

#### Third Screening Visit

At this screening session, an anesthesiologist will consult the patient. Here, they will have a complete assessment with several exams and tests including vital signs (blood pressure, heart rate, and pulse oximetry), electrocardiography, and routine bloodwork, including a complete blood count with electrolytes, creatinine, blood urea nitrogen, and liver function tests. This visit will assess the patient’s cardiovascular, hepatic, and renal health. Patients will not be able to participate if they have untreated hypertension, cardiovascular disease, renal disease, or hepatic disease.

### Procedures

Patients will first attend the screening sessions listed above, after which, if they are eligible and have provided consent, they will be assigned to one of two treatments: the combination therapy group receiving e-CBT and IV ketamine or the control group receiving no treatment for the 14 weeks during which study measures will be administered. The study coordinator will then enter participants into the study through computer-generated block randomization and will recruit them in pairs so that 10 patients will receive the experimental condition and 10 will be in the control group.

### Electronically Delivered Cognitive Behavioral Therapy

#### Overview

Participants from the combination therapy group will begin an e-CBT program, which will involve a 14-week course of TF-CBT. The content of the therapy course will mirror the in-person TF-CBT and cognitive processing therapy intervention for PTSD [36]. The format for the content of the modules and the overall structure of the therapy course and delivery platform is based on previous work by Alavi et al [37-39]. All web-based sessions will be conducted through the Online Psychotherapy Tool (OPTT), which is a secure, cloud-based web service for hosting asynchronous psychotherapy. Patients will first be introduced to their therapist, who will then email the patient a link to their weekly module that will be presented to them in the form of approximately 30 presentation slides. Each week’s module will highlight a particular topic and will include general information, an overview of skills, and homework to be completed at any time within the week. OPTT will save a patient’s progress so that they may work at their own pace, resuming when it is convenient for them. This homework will take approximately 40 minutes to complete and will be submitted within 1 week via OPTT to the therapist, who will provide personalized feedback across the same platform.

Therapist feedback will involve content that seeks to build rapport, review skills, review the content of the patient’s homework, and provide constructive feedback. A detailed explanation of this structure can be found in *Online Cognitive Behavioral Therapy: An e-Mental Health Approach to Depression and Anxiety*, a book by Alavi and Omrani [40]. Although homework and clinician feedback are considered as the main modes of communication between therapists and participants, participants can also communicate with their therapist via a secure chat function that is found directly within the OPTT. This is mainly used to let participants ask further questions about their care if anything is unclear. The OPTT technical support team will handle any technical issues and provide continuous access to the participants during the program. The patient care team (ie, the therapist and the psychiatrist) will also be able to securely communicate through the OPTT to make decisions regarding each patient’s care path. Finally, if a patient does not complete their homework for the week, they will receive 3 weekly emails, after which they will be removed from the study.

#### Web-Based Module Content

**Figure 2:**
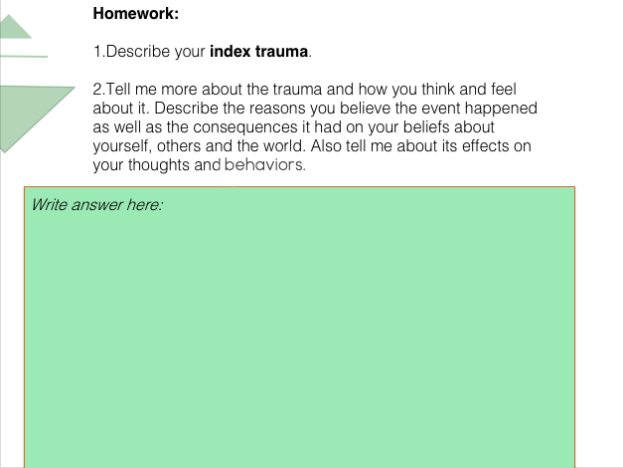
Web-based intervention example material.

The TF-CBT is focused on strategies that would be helpful in handling stress and mood problems related to the trauma experienced by patients. The program helps patients independently manage their emotions, thoughts, and behaviors. The course is specifically designed to address the need for healing from traumatic events and to facilitate recovery through trauma-informed care. Topics include stuck points, identifying events, index events, problematic thinking, challenging beliefs, safety, and trust (see Figure 2 for an example of the module content).

#### Training

All therapists are research assistants hired by the coprincipal investigator leading the web-based psychotherapy portion of the research. They will undergo training in psychotherapy and additional training from a psychiatrist on the research team before any interaction with the participants. During this training, therapists will complete feedback on practice homework, which will be reviewed by a psychiatrist on the research team to ensure an adequate quality of work. The lead psychiatrist, who is an expert in electronically delivered psychotherapy, will supervise all the therapists [40], and will review feedback before it is sent to the participants.

### Ketamine

Patients in the combination therapy group will also receive ketamine infusions at the Providence Care Hospital Ketamine Clinic. An indwelling catheter will be first placed in the antecubital vein of the nondominant arm. Patients will then be administered with an IV subanesthetic dose of ketamine hydrochloride (0.5 mg/kg) over 40 minutes. Nasal cannula oxygen may be administered, if needed, with sidestream capnometry monitoring. Pulse, blood pressure, pulse oximetry, and electrocardiography will be assessed before the start of each infusion and will be monitored throughout the infusion for adverse effects, such as an increase in blood pressure and dissociative state, which will terminate an infusion if identified. Physiological monitoring data will be recorded on a standard anesthesia record beginning 5 minutes before infusion. Participants will complete a total of 6 infusions over a 14-week period. Participants will receive 1 dose per week for the first 4 weeks, followed by 1 dose every other week for the next 4 weeks, followed by 0 doses per week for the remaining 6 weeks of the study. Side effects will be recorded before each infusion, at the end of each infusion, and 30 minutes after the end of each infusion. To reduce the chances of adverse psychological reactions, patients will be kept in a room with reduced tactile, visual, and auditory stimulations throughout the infusion and recovery period. Patients will be instructed not to operate a vehicle or heavy machinery on the day following ketamine infusion and will require a responsible adult to accompany them to their appointments. Patients and chaperones will be compensated for public transit or parking fares. Finally, patients will undergo monthly assessments with a clinician where they may report any adverse physical or psychological symptoms that arise over the course of treatment. All adverse events will be recorded, tabulated, and reported in the final publication. Table 1 summarizes the ketamine administration protocols.

**Table 1:** Summary of the ketamine administration protocol.

Characteristic	Specification
Generic product name	Ketamine
Dose (mg/kg)	0.5
Route of administration	Intravenous
Dosing schedule	Weekly×4, then biweekly×2
Ketamine treatment period	8 weeks

### Control

#### Overview

Patients in the control condition will be put on a 14-week waitlist during which they will receive regular psychiatric care, including continuing any previous treatment regimens and receiving monthly check-ups. These patients will also be assessed at the same 4 time points as patients in the experimental condition. At the end of the 14-week experimental period, patients in the control group will receive the experimental treatment.

#### Stopping Guidelines

The following conditions, if met, will necessitate a participant’s removal from the study:

1. If a participant failed to submit their e-CBT homework within 21 days of receiving their module for a given week and after receiving 3 reminders, or if a participant missed a ketamine appointment as well as their makeup appointment.
2. If a participant were to develop adverse effects from participation so that the principal investigator deems it unsafe for them to continue, such as physical or psychological adverse side effects from the ketamine (eg, allergy), or if participants develop psychologically adverse symptoms resulting from the assessments or e-CBT.
3. If a participant were to meet an exclusion criterion during the study duration.
4. If a participant withdraws consent for any reason.

Multimedia Appendix 1 provides more information on stopping guidelines and general safety procedures.

### Outcome Evaluation

#### Overview

Patient outcomes will be measured through clinical interviews and questionnaires completed at the start (baseline measurement) and end of treatment. The primary measure, covered in detail in the following section titled *Primary Outcome Measure*, will also have midway assessments at 4 and 8 weeks through treatment. All questionnaire data will be administered electronically to the patients through OPTT alongside their e-CBT sessions that week. Interviews and observational data will be collected either in person or through virtual (video) appointments.

#### Primary Outcome Measure

The primary outcome measure is the CAPS-5 interview [34]. The treatment response is defined as a 50% reduction in the participants’ scores at the end of the 14-week period as compared with their scores at baseline. Nonresponse is defined as less than a 50% reduction in scores. Remission is defined as a 75% reduction in scores, whereas relapse is defined as a temporary treatment response or remission at one or both of the halfway points with a return to nonresponse at the final assessment.

#### Secondary Outcome Measures

Secondary outcome measures will include the following:

1. The Montgomery-Asberg Depression Rating Scale to measure depression symptoms [41].
2. Columbia-Suicide Severity Rating Scale, risk assessment version to measure suicidality [42].
3. The Clinical Global Impression scale to measure a patient’s overall clinical presentation from a clinician’s perspective and to provide interrater reliability [43].
4. Sheehan Disabilities Scale to provide insight into a patient’s social and occupational functioning [44].
5. The Global Assessment of Functioning Scale provides additional insights into social and occupational function [45].

### Ethics and Data Privacy

All procedures were approved by and comply with the Queen’s University Health Sciences and Affiliated Teaching Hospitals Research Ethics Board. Multimedia Appendix 2 [1,2,4,7-15,18-24,28-33,46-54] shows the full protocol approved by the research ethics board. Each participant will be given an anonymous, unique code, with all screening and study outcome measures, including interviews, questionnaires, and observations, which will be associated with the patient’s code alone. All data will be stored as encrypted files on a secure Queen’s University server for 5 years after the study completion date.

The research team will protect the identity and confidentiality of participants to the extent permitted by the applicable laws and duty to report. Child abuse and neglect, elder abuse, and immediate physical risk to the self or others are grounds for breaching confidentiality. The identity of participants will remain completely anonymous in all future plans for knowledge dissemination, including but not limited to peer-reviewed publications, scientific presentations, grant proposals, and reports. Hard copies of consent forms and participant identities will be securely stored on-site and destroyed 5 years after study completion.

To ensure data privacy and security, OPTT was developed to comply with the Health Insurance Portability and Accountability Act, Personal Information Protection and Electronic Documents Act, and Service Organization Control-2. All servers and databases are hosted in the Amazon Web Service Canada cloud infrastructure, which is managed by Medstack (Medstack Inc) [46] to ensure that all Canadian provincial and federal privacy and security regulations are met. For privacy purposes, the OPTT will not collect any identifiable personal information or internet protocol addresses from the participants. The OPTT will only collect anonymized metadata to improve its service quality and provide advanced analytics data to the clinical team. The OPTT will encrypt all data, and no employee will have direct access to the participant data. All encrypted backups are to be kept in Amazon S3 storage, which is dedicated to Queen’s University, Kingston, Ontario, Canada.

### Data Analysis

Data will first be entered into a spreadsheet and then imported into the R data analysis software program (R Core Team) [55]. Descriptive statistics including mean, median, SD, maximum, and minimum scores for primary and secondary outcome measures, as well as demographic data, including age and sex of the participants, will be computed and reported. Box plots for descriptive statistics will also be prepared for each outcome measure, demonstrating the mean and SD across time points and experimental conditions.

The data will be tested for statistical assumptions, including normality using the Shapiro-Wilk test, skew using Pearson coefficient of skewness, kurtosis using Pearson measure of kurtosis, homogeneity of variance using Levene test, and homogeneity of covariance using Box’s M. The results of these tests will be reported later. Outliers were not extracted because of the small sample size.

If the assumptions are met, then a 2×4 mixed effects analysis of variance (*P*=.05) will be conducted to determine the main effects of the two factors, namely *time* and *condition*, as well as the interaction effect between time and condition on PTSD symptom outcome for CAPS-5 symptom severity. *Time* comprises 4 levels, including before treatment, 4 weeks through treatment, 8 weeks through treatment, and end of treatment. However, *condition* includes 2 levels, including the experimental condition and waitlist control condition. Simple main effects will be tested for time and condition, and a Bonferroni *P* value adjustment will be made for the *time* factor. Post hoc tests will be conducted on the *time* variable with a Tukey range test.

The secondary outcome measures are exploratory in nature and will be conducted with a 2×2 mixed effects analysis of variance (*P*=.05), where *time* has only 2 levels and no post hoc tests will be conducted.

This analysis will also measure the effect size, where the interaction is expected to have a large effect size (*η^2^*=0.14). Finally, all adverse events will be recorded and reported, grouped by the type of adverse event, and reported by frequency. No interim analysis was planned for this study, and participants in the control condition will only be assessed while on the waiting list and will not be assessed for any separate analyses.

## Results

The study was approved for funding in September 2020 and received ethics approval from Health Canada and the Queen’s University Health Science and Affiliated Teaching Hospitals Research Ethics Board in May 2021. The recruitment of participants was set to begin in July 2021, based on clinician referrals from outpatient psychiatry at Hotel Dieu Hospital. Recruitment will be conducted with approximately 3 participants added each month until January 2022. The study outcomes will be shared with the National Institute of Health ClinicalTrials.gov database in the summer of 2022.

## Discussion

### Principal Findings

The main anticipated findings of this study will evaluate the efficacy of a novel intervention in a previously treatment-resistant patient population. Although ketamine has been used to treat other affective disorders, very few trials have been conducted on its use for treating PTSD. In addition, ketamine and CBT have been successfully combined in the past to treat other psychiatric disorders [47], but they have never been amalgamated to treat PTSD, nor have they ever been combined with a web-based component. This study will also increase access to care and help hospitals and clinics provide patients with accessible and affordable treatment. The ketamine component of this study will improve access due to its rapid symptom relief [48] and the resulting increased patient volume. Similarly, e-CBT will improve access to treatment for patients without the time or ability to travel to an in-person clinic weekly while benefiting economically disadvantaged patients and those living in rural areas with low access to specialized care. e-CBT also provides a safe alternative to in-person therapy during the COVID-19 pandemic. Such an approach to treatment can help address lengthy wait times and the cost of mental illness in health care systems [30,31]. Finally, this study aims to contribute toward the discussion on glutamate and its role in fear extinction. An effective combined treatment would suggest that the glutamatergic system may help in facilitating fear extinction.

### Limitations and Future Directions

As this is a proof-of-concept study, the goal is to determine whether the treatment model works to reduce PTSD symptoms. Nevertheless, because of the preliminary aims of this study, there are a number of limitations. First, if the treatment successfully reduces symptoms, this protocol offers no way to determine the source of the improvement. Symptom improvement could be attributed primarily to e-CBT, ketamine, or both. A second issue is that this study does not offer insights into the posttreatment timeline for relapse, as the last assessment is immediately following treatment. Another issue is the unblinded nature of the study potentially biasing the participants as a result.

In terms of future directions, a follow-up study is needed with 40 participants and a four-arm design, including a control group receiving an active ketamine placebo and sham-CBT, a group receiving true e-CBT and placebo ketamine, a group receiving true ketamine and sham-CBT, and a group receiving both ketamine and e-CBT. Furthermore, follow-up assessments should be performed at 3 months, 6 months, and 1 year after treatment. These research design elements would allow for an investigation of the compounding effects of the two treatments and the symptom time-course posttreatment.

## Appendix

Multimedia Appendix 1 Safety parameters.

Multimedia Appendix 2 Full protocol approved by the research ethics board.

Multimedia Appendix 3 Peer review document for funding.

Multimedia Appendix 4 Funding approval letter.

Multimedia Appendix 5 CONSORT-eHEALTH checklist (V 1.6.2).

## Abbreviations

CAPS: Clinician-Administered PTSD Scale
CBT: cognitive behavioral therapy
*DSM-5*: *Diagnostic and Statistical Manual of Mental Disorders, 5th edition*
e-CBT: electronically delivered cognitive behavioral therapy
IV: intravenous
NMDA: N-methyl-D-aspartate
OPTT: Online Psychotherapy Tool
PTSD: posttraumatic stress disorder
TF-CBT: trauma-focused cognitive behavioral therapy
