# Supplementary material for: Combining Ketamine and Internet-Based Cognitive Behavioral Therapy for the Treatment of Posttraumatic Stress Disorder: Protocol for a Randomized Controlled Trial
Source: JMIR Res Protoc. 2021 Jul 20;10(7):e30334. doi: 10.2196/30334 (PMC8335614; doi:10.2196/30334)
Supplement: Multimedia Appendix 1 [file resprot_v10i7e30334_app1.pdf]

Risks and Risk Management

Potential Risks associated with Ketamine:

- *Short-term physical side effects:* nausea, vomiting, dilated pupils, changes in eyesight, inability to control eye movements, involuntary muscle movements, muscle stiffness, slurred speech, numbness, increased pressure in the eyes and brain, loss of appetite, chest pain, elevated or depressed heart rate, and elevated blood pressure, and allergic reaction.
- *Longer-term physical side effects:* On very rare occassions, patients may experience inflammation or damage to the urinary tract, and adverse effects on liver function such as elevated liver enzymes, hepatic fibrosis, or biliary ductal dilatations.
- *Short-term psychological side effects:* drowsiness, changes in colour perceptions, sound hallucinations, confusion, delirium, dissociation from body or identity, agitation, and amnesia.
- *Long-term psychological side effects:* Occassionally, patients may develop addiction to ketamine.
- Ketamine produces drowsiness for several hours after a subanesthetic dose. Patients will therefore be required to have a responsible adult accompany them to each appointment so that they may be driven home afterwards. Patients will be instructed not to operate a vehicle or heavy machinery for the rest of the day following a ketamine infusion.
- In order to reduce the chances of adverse psychological reactions, patients will be kept in a room with reduced tactile, visual and auditory stimulation. Additionally, patients will have monthly assessments with a clinician where they can report any adverse physical and/or psychological symptoms that may arise over the course of treatment.

Risks associated with online CBT

- The psychotherapy and psychological assessments will involve personal questions concerning a patient’s mental health as it pertains to PTSD. This includes questions and activities that may

involve facing a personal psychological trauma, suicidal thoughts, as well as mood and anxiety issues. These questions could theoretically be triggering and may lead to temporarily heightened symptoms. The psychiatrist on the team will keep track of all participants, may discontinue enrolment and potentially direct patients to seek additional mental-health support if they believe it to be necessary in maintaining a patient's mental health.

- With the online CBT program, the facilitator only receives and reads messages from patients once a week. Therefore, if a patient is in crisis during the week, they will be instructed not to email the facilitator, but to instead call 911, a mental health crisis line, go to the nearest emergency room, or call their family doctor.
- Participants in both experimental and control conditions will be checked on once per month by their clinician. If the clinician believes that the study is causing unreasonable psychological stress on the participant, they may require the study coordinator to remove the particular patient from the study.

Methods for Assessing, Recording, and Analyzing Safety Parameters Related to Ketamine

Pulse, blood pressure, pulse oximetry, and electrocardiography will be assessed before the start of each infusion and will be monitored throughout the infusion for adverse effects, including any of the risks associated with ketamine mentioned above. A registered nurse and psychiatrist will monitor the patients for the physical and psychological symptoms mentioned above. Identification of such risks will terminate an infusion. Physiological monitoring data will be recorded on a standard anaesthesia record beginning 5 min prior to infusion. Status will be recorded before each infusion, at the end of each infusion, as well as 30 minutes after the end of each infusion. Patients will also have monthly visits with their psychiatrist where they should

report adverse physical symptoms. The recorded side effects will be analyzed using descriptive qualitative statistics as simple frequency data and will be included in future reports.

To obtain safety reports, the overseeing psychiatrist or nurse at the ketamine clinic will provide the study coordinator with a printout of the safety report. If patients experience long-term or delayed adverse side-effects or simultaneous illnesses, patients will inform their psychiatrist, who will forward the report to the study coordinator. The study coordinator will add any adverse events onto the master spreadsheet containing all the assessment data for all participants. This information will be put in a column titled 'adverse events' in the final spreadsheet.

Acute Follow-Up to Adverse Events

In the case of severe anxiety as a result of ketamine's depersonalizing effects, patients may be given a dose of Lorazepam as a fast-acting anxiolytic (.044 mg/kg, IV or sublingual). In the rare case that a patient undergoes an anaphylactic allergic reaction, patients can receive 0.15mg I.M. of Epinephrine, as well as 8mg I.V. of dexamethasone and 25mg I.V. of diphenhydramine. The patient may also require endotrachial intubation.

The remaining short-term physical and psychological symptoms will be monitored for at least 30 minutes after the end of the infusion. If conditions persist, the psychiatrist will either send the patient to urgent care if the condition is worsening and/or generally severe, or they will send the patient home if the condition is mild, recommending that they return to hospital if the condition worsens or dial 9-1-1 if it becomes severe.

Long-Term Follow-Up to Adverse Events

For long-term adverse physical side effects of ketamine such as effects on liver and urinary function, as well as long-term adverse psychological side effects such as addiction, patients will be able to seek medical assistance at their monthly check-up with their psychiatrist, who will independently ask the patient about these long-term symptoms at the first 3 check-ups following their last ketamine appointment. Patients that present any of these long-term symptoms will be referred to appropriate medical or psychotherapeutic specialists.
