# Supplementary material for: Combining Ketamine and Internet-Based Cognitive Behavioral Therapy for the Treatment of Posttraumatic Stress Disorder: Protocol for a Randomized Controlled Trial
Source: JMIR Res Protoc. 2021 Jul 20;10(7):e30334. doi: 10.2196/30334 (PMC8335614; doi:10.2196/30334)
Supplement: Multimedia Appendix 2 [file resprot_v10i7e30334_app2.docx]

**Multimedia Appendix 2**. Full protocol approved by the research ethics board.

**Trial Title:** Combined Ketamine and eCBT Treatment for PTSD

**Date:** May 4, 2021

**Sponsor:** Queen’s University

**Name and title of the person(s) authorized to sign the protocol:** Dr. Taras Reshetukha

**Protocol Number:** 94373792536

**Version Number:** 02

**Statement of compliance:** This trial will be conducted in compliance with the protocol, GCP, and the applicable regulatory requirements as per section 6.2.5 of the ICH E6(R2).

**Name and description of the investigational product(s)**

KETALAR, dl 2-(o-chlorophenyl)-2-(methylamino) cyclohexanone hydrochloride, is a white crystalline compound, soluble in water to 20%, and is a clear and colourless solution. Aqueous solutions in use have pH range from 3.5 to 5.5. The base component is 86.7% of the salt. It is supplied as a slightly acidic (pH 3.5 - 5.5) solution for intravenous or intramuscular injection in concentrations containing the equivalent of either 10 to 50 mg ketamine base per mL and contains 0.01% Phemerol (benzethonium chloride) as a preservative. The solution is made isotonic with sodium chloride.

### **Background and Rationale**

##### Post-Traumatic Stress Disorder (PTSD) is a chronic and debilitating mental illness occurring in 3.5% of North American adults with a lifetime prevalence of 8% (APA, 2013). PTSD develops after direct or indirect exposure to a psychologically traumatic incident, leading to a host of cognitive, emotional and behavioural symptoms (APA, 2013). While most patients recover after a psychological trauma, over 30% of individuals remain chronically symptomatic (Levy, Huong and Bonanno, 2018). PTSD is a particularly refractory disorder, lasting for years, with approximately 15% of patients still displaying symptoms 10 years after a traumatic experience (Kulka et al., 1990). By some accounts, PTSD prevalence may grow as time increases post-trauma (Solomon, 1990). PTSD is also associated with high comorbidity rates, with up to 87.5% of patients with PTSD also suffering from diagnosed depression or anxiety (Perkonigg et al., 2000) and approximately 50% of patients treated for substance abuse meeting diagnostic criteria for PTSD (Brown, Stout and Mueller, 1999). Finally, patients with PTSD are at a far greater risk of attempting suicide than the general population (Wilcox, Storr, and Breslau, 2009). Taken together, these features draw attention to the urgent need of effective strategies for treating this disorder.

##### A number of empirically supported psychotherapeutic treatments are available for PTSD, with Trauma-Focused Cognitive Behavioural Therapy and Eye Movement Desensitization and Reprocessing Therapy being among the most effective (Bison et al., 2013; Roth and Fonagy, 2005). However, traditional treatments have a considerable non-response rate, ranging from 33 to 50 percent, depending on the measure (Schottenbauer et al, 2008; Bradley et al., 2005). Meanwhile, a number of pharmacotherapies have become available to treat PTSD, with SSRIs such as Paroxetine and Sertraline seeing the greatest success (Stein et al., 2006). Unfortunately, SSRI treatments simply reduce symptom severity rather than providing true symptom remission (Friedman et al., 2007). Furthermore, over 40% of patients with PTSD are non-responsive to existing medication (Stein et al., 2006). Thus, there remains a sizeable proportion of patients with PTSD that are treatment resistant. The goal of the current study will be to provide these patient with a treatment plan that could help with symptom remission or at least a greater reduction in symptoms compared to current available treatments.

##### Ketamine is a promising research avenue for treating refractory PTSD. It is primarily a glutamate antagonist at the N-methyl-D-aspartate (NMDA) receptor, and it has had considerable success in rapidly reducing symptoms of a host of affective disorders (Sanacora et al., 2017). Ketamine is thought to function by disengaging an established pattern of thought (Das et al., 2019; Girgenti et al., 2017), which in the case of PTSD would involve counteracting an impaired fear extinction by increasing a patient’s neuroplasticity towards fear learning. It is important to note that its exact mechanism of action in the treatment of emotional disorders is only partly understood. However, recent advances have generally implicated the NMDA receptor in the development of PTSD, where rodents subjected to chronic stress have elevated gene-expression for production of NMDA receptors in the ventral hippocampus in comparison to control subjects (Calabrese et al., 2012). Moreover, in humans, the prefrontal cortex and amygdala are connected by glutamatergic projections, suggesting that glutamate mediates a fear response (Del Arco and Mora, 2009). Ketamine, in turn, has had considerable success in treating refractory PTSD, where it significantly reduces symptoms in comparison to an active placebo for treatment-resistant patients (Feder et al., 2014). Ketamine has also provided a rapid reduction in suicidal ideation (Wilkenson et al., 2018; Price et al., 2009), providing an additional benefit to this novel treatment.

##### While Ketamine opens treatment options to a new patient cohort, a purely pharmacological approach would be an oversimplification of the nature of PTSD, as the disorder develops in the wake of a trauma, and cannot develop from pathological neurochemistry or neuroanatomy alone. Moreover, Ketamine’s effects wear off in under a week (Feder et al., 2014; Murrough et al., 2013), and repeated infusions can have potentially negative outcomes on cognitive and physical health in the longterm (Hassler, 2019). One potential way to prolong its effects is to capitalize on Ketamine’s role in facilitating fear extinction by combining it with psychotherapy. This is partly based on the finding that psychotherapeutic interventions have considerably more longevity than pharmacological techniques for reducing symptoms of PTSD (Merz et al., 2019). At the moment, there have been very few studies combining Ketamine and psychotherapy to treat PTSD. As of December 2020, there were 4 studies listed on the [ClinicalTrials.gov](http://clinicaltrials.gov) database investigating Ketamine used in combination with psychotherapy to treat PTSD (NCT02727998; NCT02766192; NCT04560660; NCT03960658). The results of these studies have been promising: for example in one study, a round of mindfulness-based psychotherapy successfully lengthened Ketamine’s effects from one week up to 33 days (Pradhan et al., 2018). The proposed study would therefore attempt to build on these findings by examining other types of psychotherapy in conjunction with Ketamine to treat PTSD.

##### Trauma-focused Cognitive-Behavioural Therapy (TF-CBT), as mentioned above, is a well-established form of psychotherapy used today to treat PTSD (WHO, NICE). In general, TF-CBT (and EMDR therapy) is considered among the most effective forms of psychotherapy for PTSD immediately post treatment and at follow-up (Bisson et al., 2013). However, as mentioned above, there is still a considerable proportion of PTSD patients that do not respond even to TF-CBT. One challenge associated with CBT in general is in optimizing inhibitory learning, which is inherently challenging for patients with PTSD (Craske et al., 2008; Vasterling and Hall, 2018). Ketamine treatment may address this challenge as it acts to boost neuroplasticity. Another challenge with CBT is that psychotherapy is a resource intensive form of healthcare with large costs and waiting times (Loebach and Ayoubzadeh, 2017; Wilson et al., 2016). Traditional face-to-face CBT also poses risks in light of the current COVID-19 pandemic. One variant of CBT that addresses all of these challenges is online Cognitive Behavioural therapy (eCBT), which has equivalent efficacy as face-to-face CBT for treating PTSD as seen in several meta-analyses (Sloan et al, 2010; Sijbrandij et al., 2016).

##### Thus, we hypothesize that a combination of Ketamine and eCBT would enhance both treatments, allowing eCBT to lengthen and reinforce the effects of Ketamine, while allowing Ketamine to bolster inhibitory learning in eCBT. Unfortunately, there has yet to be a study examining the interaction of Ketamine treatment with eCBT or CBT in general. Therefore, this study will investigate whether ketamine in combination with eCBT is capable of significantly reducing symptoms of PTSD in treatment-resistant patients.

###### **Objectives and Purpose**

This project strives to provide therapy to treatment-resistant PTSD patients, where eCBT lengthens and reinforces the effects of Ketamine, while Ketamine bolsters inhibitory learning in eCBT. Ketamine and CBT-type therapies have been successfully combined in the past to treat other psychiatric disorders (Krystal et al., 2019) but they have never been combined to treat PTSD nor have they ever been combined with an online component. A combined interventional approach is expected to open new therapeutic options for area ment-resistant patients. The current study will also increase access to care, helping Canadian healthcare institutions provide patients with accessible and affordable treatment. The Ketamine component of this study would improve access due to its rapid symptom relief (Zarate et al., 2006), allowing more patients to ultimately be seen. Likewise, the eCBT component would improve access to treatment for patients without the time or ability to travel to an in-person clinic, benefiting economically disadvantaged patients and those li ing in rural areas with low access to specialized care. eCBT also provides a safe alternative to in-person therapy during the COVID-19 pandemic. Such an approach to treatment can help address lengthy wait times and the costs of mental illness on the Canadian healthcare system (Loeback and Ayoubzadeh, 2017; Wilson et al., 2016). Finally, this study will draw attention to the role of Glutamate in producing fear, anxiety, and related disorders, given that Ketamine operates on the NMDA receptor. This study will therefore help provide further understanding of drugs operating on Glutamatergic receptors and their uses in treating affective disorders.

**Study Design Overview**

The current proposed study will involve a parallel design. This will involve two treatment groups, one receiving the experimental treatment, and the other being put on a waitlist, receiving treatment as usual. Both groups will be measured before, during and after treatment. See the diagram below for a schematic:


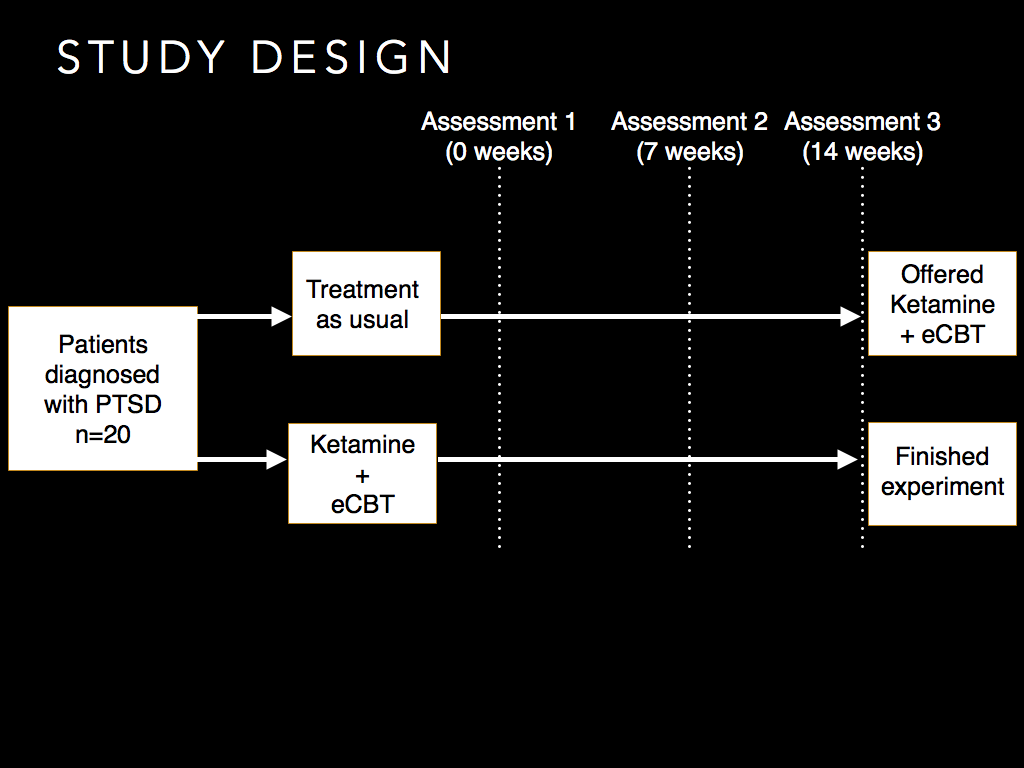
Figure 1: Schematic of Experimental Design and Course of Events

Please note that the study will follow a 23 - 35 week timeline, including an 8-week period of stable treatment required of all patients before recruitment (serving as a prerequisite for inclusion), 3 weeks of screening, 14 weeks of experimental treatment (or waitlist treatment), and 14 weeks of post-experiment treatment (for patients in the waitlist condition).

**Participant Recruitment, Consent, and Screening**

Patients (N=20) diagnosed with PTSD will be referred to this study by their admitting physician at Hotel Dieu Hospital in the Adult Mental Health Program. A power analysis using a moderate to large effect size (*f* = .349), based on the effect size in previous work (Pradham et al., 2018), demonstrates that a sample of 20 participants divided into 2 treatment groups, each measured on 3 occasions yields a power of .918. As this project is still in the pilot phase, we would like a power of at least .9 in order to have a low chance of a false negative. The admitting physicians will first be given flyers containing a brief description of the study with inclusion/exclusion criteria and contact information. They will then provide incoming patients that match the basic criteria with a copy of the flyer, informing them of this study. Patients that are interested in participating will contact the study coordinator who will call the patient to conduct a prescreening interview, asking basic questions to determine if the patient may be eligible. If the patient passes this interview, the study coordinator will book the patient for an assessment with a psychiatrist on the team for confirmation of a diagnosis of PTSD, and to screen patients for inclusion/exclusion criteria. If the patient is eligible and agreeable to participate, they will be entered into the study.

Each patient will then be scheduled for a screening session with the Mini International Neuropsychiatric Interview, conducted over the phone by a trained research assistant. Each patients will then be randomly assigned either to the control treatment group (n=10), or the experimental combination treatment group (n=10).

At this screening session, the patient will be consulted with an anesthesiologist, and will be given a number of assessments, including vital signs (blood pressure, heart rate, and pulse oximetry), electrocardiogram, as well as routine bloodwork, including a complete blood count with electrolytes, creatinine, blood urea nitrogen, and liver function tests. This visit will assess the patient’s cardiovascular, hepatic and renal health. Urine pregnancy tests will also be administered to women of childbearing potential. Patients will not be able to participate if they have untreated hypertension, cardiovascular, renal, or hepatic disease, or if they are pregnant.

If the patient passes all three screening assessments and is still interested, they will then be booked for an appointment with the study coordinator for an information session where patients have the option to give consent. At this meeting the study coordinator will provide complete information concerning the rationale, procedures, as well as potential risks and benefits to participating in this study, and will answer any questions the patient might have. Patients will be informed that they can withdraw from the study at any point without penalty and will also be informed that no deception or compensation will be provided.

Data Storage:

Each participant will be given an anonymous, unique code, and all screening/study outcome measures including interviews, questionnaires, and observations will be associated with the patient's code alone. The results of all clinician administered assessments will be entered by the clinician onto a password protected spreadsheet accessible only to researchers on the team. Participant data will be associated with each participant’s unique anonymous code. A separate password protected spreadsheet will contain information associating each participant’s name and contact information with their unique code. Only the research team will know the passwords for the 2 spreadsheets. All screening and treatment outcome data and contact information will be stored on separate password protected spreadsheets, both of which will be stored on the Online Psychotherapy Tool (OPTT). To ensure data privacy and security, OPTT was developed to comply with the Health Insurance Portability and Accountability Act, Personal Information Protection and Electronic Documents Act, and Service Organization Control-2. All servers and databases are hosted in the Amazon Web Service Canada cloud infrastructure, which is managed by Medstack (Medstack Inc), to ensure that all Canadian provincial and federal privacy and security regulations are met. For privacy purposes, the OPTT will not collect any identifiable personal information or internet protocol addresses from participants. The OPTT will only collect anonymized metadata to improve its service quality and provide advanced analytics data to the clin-ical team. All data will be encrypted by the OPTT, and no employee will have direct access to participant data. All encrypted backups are to be kept in the Amazon S3 storage that is dedicated to Queen’s Univer-sity, Kingston, Ontario, Canada. All data will be stored on OPTT for 25 years after the study completion date. The file tracing participant names and contact information with their unique code **will be destroyed at the end of the data collection period.**

Participant identity and confidentiality will be protected by the research team to the extent permitted by the applicable laws and/or duty to report. Child abuse/neglect, elder abuse, and immediate physical risk to self or others will be grounds to breach confidentiality. As a final note, the identity of participants will remain completely anonymous in all future plans for knowledge dissemination, including but not limited to peer reviewed publications, scientific presentations, grant proposals, and reports.

**Inclusion Criteria**

1) Provide oral consent;

2) Patients will be 18-65 years of age at the start of the study;

3) Diagnosed with PTSD by a psychiatrist on the team using the Clinician Administered PTSD Scale (CAPS-5) with a score of at least 50 on the CAPS-5 with the required distribution of symptoms across subcategories as outlined in the DSM-5 so as to qualify at least as moderate presentation;

4) Treatment resistant so as to justify an experimental treatment method, where they will have received at least 2 different types of prior treatment, including any combination of SSRIs, SNRIs or TFCBT, and both treatments will have produced less than a 50% reduction in the participant’s symptoms;

5) On stable treatment for at least 8 weeks prior to screening, with no alterations to treatment regimen;

6) If a female of childbearing potential, using an effective method of contraception;

7) Able to speak and read in English, and having consistent and reliable access to the internet;

8) Agrees to adhere to the study protocol.

**Exclusion Criteria**

1) Prior hypersensitivity/allergy to ketamine;

2) Hypomanic/manic episodes, bipolar disorder, acute psychosis, and/or schizophrenia;

3) History of opioid or barbiturate use disorder, current use of opioids or barbiturates, or treatment with Naltrexone;

4) Currently pregnant, postpartum, or breastfeeding;

5) Untreated or inadequately controlled hypertension and/or cardiovascular disease, cerebrovascular accident, or severe cardiac decompensation

6) Elevated intracranial pressure;

7) Upper respiratory infection;

7) Significant renal or hepatic disease;

8) Antisocial personality disorder and/or active homicidal ideation.

A psychiatrist on the team will assess patients for all inclusion-exclusion criteria using self-report measures. A physician will also complete a physical exam, assess vital signs and request blood-work as well as ECG results prior to the first treatment of Ketamine. Patients that do not meet all the inclusion/exclusion criteria or with signs of cardiovascular pathology will not be able to participate.

**Procedure**

Patients will have been receiving stable treatment, with treatments being added or removed from their regimen for at least 8 weeks at the screening session, the reason being that this is the length of time needed for SSRIs to take full effect, thereby ensuring that changes in basic care are not confounded with experimental treatment effects. Patients will first be screened for the inclusion/exclusion criteria listed above. Next, they will be entered into the study through block randomization by the study coordinator using a pre-made, computer-generated random condition order. As patients are recruited, they will be assigned a condition based on the predetermined randomization order with 10 patients receiving the experimental condition involving eCBT and IV Ketamine, and the other 10 being put in the control condition, receiving no treatment for the 14 weeks during which study measures will be administered.

Participants from the combination therapy group will enrol in an online CBT treatment program. Weekly sessions of Trauma-Focused CBT (TF-CBT) will be remote and asynchronous, taking place through the Online PsychoTherapy Tool (OPTT), an online platform designed by one of the co-investigators (Dr. Nazanin Alavi). The content and format of each weekly online session will be designed to mirror the in-person TF-CBT intervention, and will be presented to the patient in the form of approximately 30 presentation slides (similar to powerpoint) each week. These 14 sessions, along with assignments, will be completed through the fully secure online environment where clinicians can interact with each other and their patients. Each week, the patient’s therapist will send them a session containing slides that highlight a particular topic, that includes general information, an overview of skills, and homework that is to be completed within the week. This homework will be submitted directly through OPTT to the therapist who will provide personalized feedback across the same platform. Only the patient and therapist will have the username and password for the patient’s account on OPTT.

Patients in the combination therapy group will also receive ketamine infusions. Participants will first undergo an overnight fast, after which an indwelling catheter will be placed in the antecubital vein of the non-dominant arm. Patients will be given a subanesthetic dose of 0.5 milligrams of Ketamine for every kilogram that they weigh. At this dose, patients remain conscious but still experience the therapeutic benefits. Nasal cannula oxygen will be administered with side-stream capnometry monitoring. Pulse, breathing, blood pressure, pulse oximetry, and electrocardiography will be assessed before the start of each infusion and will be monitored throughout the infusion until 30 minutes after the end of each infusion. Any adverse effects, such as an increase in blood pressure, dissociative state, etc., will terminate an infusion if identified. Participants will complete a total of 6 infusions over the 14-week period, each one lasting 40 minutes. Participants will receive 1 dose a week for the first 4 weeks, followed by 1 dose every other week for the next 4 weeks, followed by 0 doses a week for the remaining 4 weeks of the study. Patients will be instructed not to operate a vehicle or heavy machinery on the day following ketamine infusion. The following chart summarizes the Ketamine treatment in this study.

Figure 2: Summary of Ketamine Dosage Information

| Generic Product Name | Ketamine |
| --- | --- |
| Dose | .5 mg/kg |
| Dosing Schedule | 1 dose per week for first 4 weeks of treatment, followed by 1 dose every other week for next 4 weeks of treatment, followed by 0 doses per week for last 4 weeks of treatment |
| Route of Administration | Intravenous |
| Treatment Period | 14 weeks for both treatment arms. Follow up will be in the form of the patient’s scheduled monthly meetings with their psychiatrist. |

Patients in the control condition will be put on a 14-week waitlist during which time they will receive regular psychiatric care including continuing any treatment regimens they are already on, and receiving monthly check-ups. These patients will also be assessed at the same 3 time points for control data. At the end of the 14-week experimental period, patients in the control condition will receive the experimental treatment (eCBT and Ketamine).

**Treatment Outcome**

Patient outcome will be measured through clinical interviews and questionnaires, completed at the start (baseline measurement), midway-point, and end of the 14-week experimental period. All questionnaire data will be administered electronically to the patients through OPTT alongside their eCBT session that week. Interviews and observational data will be collected either in person or by zoom. The primary outcome measures will be the CAPS-5 interview. Treatment response will be defined as a 50% reduction in the participant’s scores at the end of the 14-week period in comparison to the participant's scores at baseline. Remission will be defined as a 75% reduction in scores while relapse will be defined as temporary treatment response or remission at the halfway point with a return to non-response at the final assessment.

Secondary outcome measures will include the Montgomery Asberg Depression Rating Scale (MADRS), the Columbia-Suicide Severity Rating Scale, risk assessment version (C-SSRS), Clinical Global Impression (CGI), Sheehan Disabilities Scale (SDS), and the Global Assessment of Functioning Scale (GAF).

**Statistical Analysis**

Data (i.e. questionnaire scores) will be collected and descriptive statistics including mean, standard deviation, maximum, and minimum scores for primary and secondary outcome measures will be computed. The score-distributions will then be compared using a 2 x 3 factorial analysis of variance (ANOVA) to determine the interaction of treatment group and time on symptom outcomes, where the treatment condition is hypothesized to have a greater reduction in scores over time than the waitlist-control condition. Effect size will also be measured in the analysis. Finally, all adverse events will be recorded and reported grouped by adverse event-type and reported by frequency. There is no interim analysis planned for this study.

**Risks and Risk Management**

Potential Risks associated with Ketamine:

- *Short-term physical side effects:* nausea, vomiting, dilated pupils, changes in eyesight, inability to control eye movements, involuntary muscle movements, muscle stiffness, slurred speech, numbness, increased pressure in the eyes and brain, loss of appetite, chest pain, elevated or depressed heart rate, and elevated blood pressure, and allergic reaction.
- *Longer-term physical side effects:* Inflammation and damage to the urinary tract, and adverse effects on liver function such as elevated liver enzymes, hepatic fibrosis, and biliary ductal dilatations.
- *Short-term psychological side effects:* drowsiness, changes in colour perceptions, sound hallucinations, confusion, delirium, dissociation from body or identity, agitation, and amnesia.
- *Long-term psychological side effects:* Addiction to Ketamine.
- Ketamine produces drowsiness for several hours after a subanesthetic dose. Patients will therefore be required to have a responsible adult accompany them to each appointment so that they may be driven home afterwards. Patients will be instructed not to operate a vehicle or heavy machinery for the rest of the day following a Ketamine infusion.
- In order to reduce the chances of adverse psychological reactions, patients will be kept in a room with reduced tactile, visual and auditory stimulation. Additionally, patients will have monthly assessments with a clinician where they can report any adverse physical and/or psychological symptoms that may arise over the course of treatment.
- Due to the risks to an embryo/fetus associated with ketamine, women of childbearing potential will be required to take a urine pregnancy test at the ketamine screening appointment. If they have a positive test, they will not be eligible to participate.

Risks associated with online CBT:

- The psychotherapy and psychological assessments will involve personal questions concerning a patient’s mental health as it pertains to PTSD. This includes questions and activities that may involve facing a personal psychological trauma, suicidal thoughts, as well as mood and anxiety issues. These questions could theoretically be triggering and may lead to temporarily heightened symptoms. The psychiatrist on the team will keep track of all participants, may discontinue enrolment and potentially direct patients to seek additional mental-health support if they believe it to be necessary in maintaining a patient’s mental health.
- With the online CBT program, the facilitator only receives and reads messages from patients once a week. Therefore, if a patient is in crisis during the week, they will be instructed not to email the facilitator, but to instead call 911, a mental health crisis line (Kingston and Frontenac 24/7 Mental Health Crisis Line: 613-544-4229), go to the nearest emergency room, call their family doctor, or contact the Providence Care Hospital switch board at 613-544-4900 and ask to see the 24-hour on-call psychiatrist.
- Participants in both experimental and control conditions will be checked on once per month by their clinician. If the clinician believes that the study is causing unreasonable psychological stress on the participant, they may require the study coordinator to remove the particular patient from the study.

Concerning the safety of eCBT, a systematic review from August 2019 assessing the acceptability of online CBT for PTSD using 10 studies with 720 participants found that adverse events for participants were reported in 2 studies, with one study finding 2 participants reporting a clinically significant increase in depression symptoms post-treatment, with one of these individuals also reporting a clinically significant increase in anxiety symptoms. However, both of these individuals had also experienced the death of an immediate family member during treatment making it difficult to attribute the increase in symptoms to the clinical intervention. The other study reporting adverse events found three individuals reported clinically significant increases in anxiety at post-treatment. No studies reported increased PTSD symptoms from baseline to last available follow-up. This data is found in Simon et al., 2019.

Methods for Assessing, Recording, and Analyzing Safety Parameters

Pulse, breathing rate, blood pressure, pulse oximetry, and electrocardiography will be assessed before the start of each infusion and will be monitored throughout the infusion for adverse effects, including any of the risks associated with Ketamine mentioned above. A registered nurse and psychiatrist will be monitoring the patients for the physical and psychological symptoms mentioned above. Identification of such risks will terminate an infusion. Physiological monitoring data will be recorded on a standard anaesthesia record beginning 5 min prior to infusion. Side effects will be recorded before each infusion, at the end of each infusion, as well as 30 minutes after the end of each infusion. Patients will also have monthly visits with their psychiatrist where they should report adverse physical symptoms. The recorded side effects will be analyzed using descriptive qualitative statistics as simple frequency data, and will be included in future reports.

To obtain safety reports, the overseeing psychiatrist or nurse at the Ketamine clinic will provide a printout of the report to the study coordinator. If patients experience long-term or delayed adverse side-effects or simultaneous illnesses, patients will inform their psychiatrist, who will forward the report to the study coordinator. The study coordinator will then copy any adverse events onto the master spreadsheet containing all the assessment data for all participants. This information will be put in a column titled ‘adverse events’. The hard copy will then be shredded.

**Follow-Up to Adverse Events**

Rescue Medication/Acute Follow-Up:

In the case of severe anxiety as a result of Ketamine's depersonalizing effects, patients may be given a dose of Lorazepam as a fast-acting anxiolytic (.044 mg/kg, IV or sublingual). In the rare case that a patient undergoes an anaphylactic allergic reaction, patients can receive .15mg I.M. of Epinephrine, as well as 8mg I.V. of dexamethasone and 25mg I.V. of diphenhydramine. The patient may also require intratrachial intubation.

The remaining short-term physical and psychological symptoms will be monitored for at least 30 minutes after the end of the infusion. If conditions persist, the psychiatrist will either send the patient to urgent care if the condition is worsening and/or generally severe, or they will send the patient home if the condition is mild, recommending that they return to hospital if the condition worsens or dial 9-1-1 if it becomes severe.

Long-Term Follow-Up:

For long-term adverse physical side effects of Ketamine such as effects on liver and urinary function, as well as long-term adverse psychological side effects such as addiction, patients will be asked questions about these potential symptoms at their monthly check-up with their psychiatrist, who will ask the patient about symptoms related to these potential long-term side-effects at the first 3 check-ups following their last Ketamine dose. Patients that present any of these long-term symptoms will be referred to appropriate medical or psychotherapeutic specialists. Female patients of childbearing potential will also be required to be taking medically acceptable birth control throughout the ketamine portion of the study (the first 6 weeks). The nursing staff will ensure that these patients are taking medically acceptable birth control prior to each ketamine infusion.

**SUSAR Statement:** Any and all serious or unexpected adverse drug reactions will be reported to Health Canada as per C.05.014 (1) of the FDR.

**Withdrawing Participants**

Premature Withdrawal and Discontinuation Criteria:

Participants may withdraw from future participation and may also withdraw their data at any time throughout the study without providing a reason and without affecting current or future medical care or health insurance.

Researchers may also remove participants from the study for the following reasons:

1. If a participant fails to submit their eCBT homework within 21 days of receiving their module for a given week and after receiving 3 reminders, or if a participant misses a Ketamine appointment as well as their makeup appointment.
2. If a participant were to develop adverse effects from participation that the principal investigator deems unsafe for them to continue, such physical or psychological adverse side effects from the Ketamine (e.g. allergy), or if participants develop psychologically adverse symptoms as a result of the assessments or eCBT.
3. If a participant were to meet an exclusion criterion during the study duration (ex. pregnancy)
4. If a participant were to have any changes to their medication or treatment regimen outside of the experiment during the 14 week period.

All the discontinuation criteria listed above are applicable only to individual participants. Additionally, all of the above require a patient to be removed from the entire study. If a participant meets any of the above, their data will immediately be archived and they will not be included in any further assessments. Participants will be notified of these measures immediately.

Details concerning subject withdrawal procedures:

1. *Type and timing of data to be collected for withdrawn subjects:* For subjects that are withdrawn from the study, no future data will be collected from them, and all data collected before the time of withdrawal will be archived for 25 years, however, it will not be used for any future analyses.
2. *Subject replacement*: Participants will be screened in pairs and randomly assigned to one of two conditions so that one participant will be streamed for each of the two conditions. There will thus always be a waitlist with at least one patient scheduled for each condition. If a patient must be withdrawn, the patient occupying the spot on the waitlist for that condition will begin treatment. This allows for replacement while ensuring randomization as well as the correct number of patients in each condition. If a patient in the last pair drops out (once there is no longer a waitlist for each condition), an additional participant will need to be recruited and screened. In this case, the study coordinator will inform the psychiatrists on the research team and the program director at the AMHP of the situation. The AMHP and research team (sans study coordinator) will proceed to conduct a blind screening, where the study coordinator will deliberately withhold from the others the condition to which this participant will need to be assigned. The psychiatrists will then administer BOTH the CAPS-5 and the MINI screening for the new participant. Once their eligibility is confirmed, they will be passed along to the study coordinator who will enter them into the appropriate condition.
3. *Follow-up for subjects withdrawn from trial treatment*: Participants that have been withdrawn for discontinuation criterion 1) and not for any of the others will be allowed to continue receiving the treatment if they choose. All participants will continue receiving monthly follow-up sessions with their psychiatrist after being withdrawn from the study where they should report any adverse symptoms that may be a result of the treatment in this study.

**Direct Access to Source Data Statement:** The investigators as well as Queen’s University will permit trial-related monitoring, audits, IRB/IEC review, and regulatory inspection(s) from Health Canada, providing direct access to source data/documents.

**References**

Abdallah, C.G., Averill, L.A. Akiki, T.J., Raza, M. Averill, C.L., Gomaa, H., Adikey, A., Krystal, J.H. (2013). The Neurobiology and Pharmacotherapy of Posttraumatic Stress Disorder *Annual Review of Pharmacology and Toxicology,* 5(91), 171-189

Alavi, Nazanin, et al. "Cognitive Behavioural Therapy through PowerPoint: Efficacy in an Adolescent Clinical Population with Depression and Anxiety." *International Journal of Pediatrics*, vol. 2018, 2018. *Gale Academic OneFile*, <https://link.gale.com/apps/doc/A583381090/AONE?> u=queensulaw&sid=AONE&xid=89625cd1..

American Psychiatric Association. Diagnostic and statistical manual of mental disorders. 5. Arlington, VA: Author; 2013.

Averill LA, Purohit P, Averill CL, Boesl MA, Krystal JH, Abdallah CG. (2017). Glutamate dysregulation and glutamatergic therapeutics for PTSD: Evidence from human studies. *Neurosci Lett 649*:147–55.

Bisson, J.I., Roberts, N.P., Andrew, M., Cooper, R., Lewis, C.C. (2013). Psychological Therapies for Chronic Post-Traumatic Stress Disorder (PTSD) in Adults. *Cochrane library* *2015* (8), CD003388–CD003388. https://doi.org/10.1002/14651858.cd003388.pub4.

Bradley, R., Greene, J., Russ, E., Dutra, L., &Westen, D. (2005). A multidimensionalmeta– analysis of psychotherapy for PTSD.American Journal of Psychiatry, 162,214–227.

Brown PJ, Stout RL, Mueller T. (1999). Substance use disorder and post-traumatic stress disorder comorbidity: Addiction and psychiatric treatment rates. *Psychology of Addictive Behaviors, 13*(2):115–122.

Calabrese F, Guidotti G, Molteni R, Racagni G, Mancini M, et al. (2012). Stress-Induced Changes of Hippocampal NMDA Receptors: Modulation by Duloxetine Treatment. *PLoS ONE 7*(5): e37916. doi:10.1371/journal.pone.0037916

Chan, S., Li, L., Torous, J. *et al.* Review of Use of Asynchronous Technologies Incorporated in Mental Health Care. *Curr Psychiatry Rep* 20, 85 (2018). <https://doi-org.proxy.queensu.ca/10.1007/> s11920-018-0954-3

Costi S, Soleimani L, Glasgow A, et al. Lithium continuation therapy following ketamine in patients with treatment resistant unipolar depression: a randomized controlled trial. Neuropsychophamacology. 2019. Epub ahead of print.

Craske MG, Kircanski K, Zelikowsky M, et al. Optimizing inhibitory learning during exposure thera py. Behav Res Ther 2008;46(1):5–27.

Das, R.K., Gale, G., Walsh, K. et al. Ketamine can reduce harmful drinking by pharmacologically rewriting drinking memories. Nat Commun 10, 5187 (2019). https:// doi.org/10.1038/s41467-019-13162-w

Del Arco A., Mora F. (2009). Neurotransmitters and prefrontal cortex-limbic system interactions: Implications for plasticity and psychiatric disorders. *Journal of Neural Transmission 116*(8): 941–952. doi: <http://dx.doi.org.myaccess.library.utoronto.ca/10.1007/10.1007/> s00702-009-0243-8.

de Kleine, R.A., Rothbaum, B.O., and van Minnen, A. (2013). Pharmacological enhancement of expo sure-based treatment in PTSD: a qualitative review, European Journal of Psychotraumatology, 4:1, DOI: [10.3402/ejpt.v4i0.21626](https://doi.org/10.3402/ejpt.v4i0.21626)

Feder A, Parides MK, Murrough JW, et al. (2014). Efficacy of Intravenous Ketamine for Treatment of Chronic Posttraumatic Stress Disorder: A Randomized Clinical Trial. *JAMA Psychiatry, 71*(6):681–688. doi:10.1001/jamapsychiatry.2014.62

Fonagy, P., Roth, A., and Higgitt, A. (2005). Psychodynamic psychotherapies: Evidence-based practice and clinical wisdom. *Bulletin of the Menninger Clinic 69*(1), 1-58.

Friedman, M.J., Marmar, C.R., Baker, D.G., Sikes, C.R., and Farfel, G.M. (2007). Randomized, double-blind comparison of sertraline and placebo for post traumatic stress disorder in a department of veterans affairs setting. *Clin Psychiatry 68*:5, 711-720.

Galatzer-Levy IR, Huang SH, Bonanno GA. Trajectories of resilience and dysfunction following potential trauma: A review and statistical evaluation. *Clin Psychol Rev*. *63*, 41-55. doi:10.1016/j.cpr.2018.05.008

Girgenti, M. J., Ghosal, S., LoPresto, D., Taylor, J. R., & Duman, R. S. (2017). Ketamine accelerates fear extinction via mTORC1 signaling. Neurobiology of disease, 100, 1–8. https://doi.org/10.1016/j.nbd.2016.12.026

Hasler, G. Toward Specific Ways to Combine Ketamine and Psychotherapy in Treating Depression. *CNS spectrums* 2019, *25* (3), 1–3. <https://doi.org/10.1017/s1092852919001007>.

Ivan Ezquerra-Romano, I., Lawn, W., Krupitsky, E., & Morgan, C. (2018). Ketamine for the treatment of addiction: Evidence and potential mechanisms. *Neuropharmacology*, *142*, 72–82. https:// doi.org/10.1016/j.neuropharm.2018.01.017

Kilpatrick, D. G., Resnick, H. S., Milanak, M. E., Miller, M. W., Keyes, K. M., & Friedman, M. J. (2013). National estimates of exposure to traumatic events and PTSD prevalence using DSM-IV and DSM-5 criteria. Journal of traumatic stress, *26*(5), 537–547. <https://doi.org/> 10.1002/jts.21848

Krystal JH, Abdallah CG, Sanacora G, et al. Ketamine: a paradigm shift for depression research and treatment. Neuron. 2019;101(5): 774–778.

Kulka RA, Schlenger WE, Fairbank JA, et al. Trauma and the Vietnam War Generation: Report of Findings From the National Vietnam Veterans Readjustment Study. New York, NY: Brunner/Mazel; 1990.

Loebach, R. and Ayoubzadeh, S. (2017). Wait times for psychiatric care in Ontario. *UWOMJ 86*:2, 48-50.

Merz J, Schwarzer G, Gerger H. Comparative Efficacy and Acceptability of Pharmacological, Psy chotherapeutic, and Combination Treatments in Adults With Posttraumatic Stress Disorder: A Network Meta-analysis. *JAMA Psychiatry.* 2019;76(9):904–913. doi:10.1001/jamapsychiatry. 2019.0951

Murrough, J.W., Iosifescu, D.V., Chang, L.C., Al Jurdi, R.K., Green, C.E., Perez, A.M., Iqbal, S., Pillemer, S., Foulkes, A., Shah, A., Charney, D.S., Mathew, S.J., 2013. Antidepressant efficacy of ketamine in treatment-resistant major depression: a two-site randomized controlled trial *Am. J. Psychiatry, 170*, 1134-1142

National Institute of Clinical Excellence (NICE). *Post*‐*traumatic Stress Disorder (PTSD). The Management of PTSD in Adults and Children in Primary and Secondary Care*. London: NICE; 2005.

Perkonigg, A., Kessler, R. C., Storz, S., & Wittchen, H. (2000). Traumatic events and post‐ traumatic stress disorder in the community: prevalence, risk factors and comorbidity. *Acta Psychiatrica Scandinavica, 101*(1), 46–59. https://doi.org/10.1034/j. 1600-0447.2000.101001046.x

Pradhan B, Mitrev L, Moaddell R, Wainer IW. d-Serine is a potential biomarker for clinical response in treatment of post-traumatic stress disorder using (R,S)-ketamine infusion and TIMBER psy chotherapy: a pilot study. Biochim Biophys Acta, Proteins Proteomics. 2018;1866:831–9. https://doi-org.proxy.queensu.ca/10.1016/j.bbapap.2018.03.006.

Price, R. B., Nock, M. K., Charney, D. S., & Mathew, S. J. (2009). Effects of intravenous ketamine on explicit and implicit measures of suicidality in treatment-resistant depression. *Biological psychiatry*, *66*(5), 522-526.

Sanacora G, Frye MA, McDonald W, et al. A Consensus Statement on the Use of Ketamine in the Treatment of Mood Disorders. *JAMA Psychiatry.* 2017;74(4):399–405. doi:10.1001/jamapsy chiatry. 2017.0080

Schottenbauer, M.A., Glass, C.R., Arnkoff, D.B., Tendick, V., and Gray, S.H. (2008). Nonresponse and Dropout Rates in Outcome Studies on PTSD: Review and Methodological Considerations. *Psychiatry: Interpersonal and Biological Processes* 71(2), 134-168.

Sijbrandij, M.; Kunovski, I.; Cuijpers, P. Effectiveness of Internet Delivered Cognitive Behavioural Therapy for Post-Traumatic Stress Disorder: a Systematic Review and Meta-analysis: Review: Internet-Delivered CBT for PTSD. *Depression and anxiety* **2016**, *33* (9), 783–791. https:// [doi.org/10.1002/da.22533](http://doi.org/10.1002/da.22533).

Simon, N., McGillivray, L, Roberts, N.P., Barawi, K., Lewis, C.E., & Bisson, J.I. (2019). Acceptability of internet-based cognitive behavioural therapy (i-CBT) for post-traumatic stress disorder (PTSD): a systematic review, *European Journal of Psychotraumatology, 10*:1.

Sloan DM, Gallagher MW, Feinstein BA, et al. Efficacy of telehealth treatments for posttraumatic stress‐related symptoms: a meta‐analysis. *Cogn Behav Ther* 2011;40(2):111–125.

Solomon Z. (1993). Twenty years after the Yom Kippur War: the belated recognition of war- induced psychic trauma. *Isr J Psychiatry Relat Sci. 30*(3):128-129.

Stein D.J., Ipser, J.C., Seedat, S., Sager, C., Amos, T. (2006). Pharmacotherapy for post traumatic stress disorder (PTSD). *Cochrane Database of Systematic Reviews*, 1. Art. No.: CD002795. 10.1002/14651858.CD002795.pub2.

Van Ameringen, M., Mancini, C., Patterson, B., Boyle, M.H. (2006). Post-traumatic stress disorder in Canada. *CNS Neurosci Ther. 14*: 171-181.

Vasterling, J.J., Arditte Hall, K.A. Neurocognitive and Information Processing Biases in Posttraumatic Stress Disorder. Curr Psychiatry Rep 20, 99 (2018). <https://doi-org.proxy.queensu.ca/10.1007/> s11920-018-0964-1

WHO. *Guidelines for the Management of Conditions Specifically Related to Stress*. Geneva, Switzerland: WHO; 2013.

Wilcox, H. C., Storr, C. L., & Breslau, N. (2009). Posttraumatic stress disorder and suicide attempts in a community sample of urban american young adults. Archives of general psychiatry, 66(3), 305–311. https://doi.org/10.1001/archgenpsychiatry.2008.557

Wilkinson ST, Ballard ED, Bloch MH, et al. (2018). The effect of a single dose of intravenous keta mine on suicidal ideation: a systematic review and individual participant data meta-analysis. *Am J Psychiatry, 175*(2):150-158.

Zarate CA, Jr, Singh JB, Carlson PJ, Brutsche NE, Ameli R, Luckenbaugh DA, et al. (2006). A randomized trial of an N-methyl-D-aspartate antagonist in treatment-resistant major depression. *Arch Gen Psychiatry, 63*:856–864.
