# Supplementary material for: Combining Ketamine and Internet-Based Cognitive Behavioral Therapy for the Treatment of Posttraumatic Stress Disorder: Protocol for a Randomized Controlled Trial
Source: JMIR Res Protoc. 2021 Jul 20;10(7):e30334. doi: 10.2196/30334 (PMC8335614; doi:10.2196/30334)
Supplement: Multimedia Appendix 3 [file resprot_v10i7e30334_app3.pdf]

Queen's University

Queen's University Department of Psychiatry Internal Research Grant Review

Title of Grant: Large grant-stream of $20,000

Primary Investigator: Taras Reshetukha

Instructions:
Assess per the adjudication scale and list 3-5 strengths and/or weaknesses in point form in the space provided.

**Adjudication Scale**	
**4.5 – 4.9** Exceptional	**3.0-3.4** Very good however needs revision to be fundable
**4.0-4.4** Outstanding	**2.5-2.9** Needs major revision
**3.5-3.9** Excellent, may still require revision	**0.0-2.4** Seriously flawed

**Originality/Scientific Merit:**	**Adjudication Score: 4.4**
Comments: Trauma and resultant Post-traumatic stress disorder (PTSD) development is an important health concern that is associated with high degrees of disability and impairment of social wellness. Therefore, effective treatments are much needed for PTSD. Currently, antidepressant and cognitive-behavioral therapy have the greatest evidence base but still do not yield a remission of PTSD symptoms in many patients. In this proposal a combined use of ketamine and e-CBT will be employed in 40 patients diagnosed with PTSD. 20 control patients will be randomly assigned to a standard treatment and 20 patients will be assigned to the experimental combination therapy group. Patients in the control group will be offered the combination therapy after the first 12 weeks, which will allow for an additional observation of the efficacy of the combination therapy.<br><br>This is a timing and innovative proposal that is expected to yield rapid results on the efficacy of the combined use of ketamine and e-CBT for PTSD.	
**Strengths:**	**Weaknesses:**

1) Excellent and timing proposal that will provide rapid information on the combined approach using ketamine and e-CBT for PTSD<br>2) Team with expertise to successfully conduct the study<br>3) Ketamine treatment for MDD and bipolar as one of the research strengths at Queen's.	1) The proposal could be clear in general. Abstract should be more comprehensive, with brief introduction, hypothesis, aims and objectives, as well as a last paragraph describing the impact. The Introduction could present a better flow of ideas and the hypothesis should be clearly stated. This is such an important project that is easy to sell and there is room for improvements in each section when preparing a CIHR proposal. It is important to spell it out to the reviewers why this work is so unique and important. Impact section could be improved by putting it in context with other similar combined approaches (specifically ketamine + psychotherapy-augmentation) tested or currently being tested in depression, bipolar, and of course PTSD.<br><br>2) In methodology it is not clear if after the completion of the study, and after control patients complete treatment, the results will be analyzed altogether.

Impact and Research Applicant(s)

**Adjudication Scale (Research impact and Research Applicant(s))**	
**4.5–4.9** Extremely significant impact appropriate team	**3.0-3.4** Moderate impact and/or poor team
**4.0-4.4** Very significant impact ,appropriate team	**2.5-2.9** Limited impact and/or poor team
**3.5-3.9** Significant impact, appropriate team	**0.0-2.4** Negligible impact and/or poor team

**Potential Impact and Team:**	**Adjudication Score: 4.4**
**Strengths:**	**Weaknesses:**

1) PI has appropriate training and expertise to recruit and assess patients and to oversee study progress.<br><br>2) Team with complementary expertise to develop the proposal. Dr. Vasques experience in treating mood disorders patients with ketamine. This is such an important.<br><br>3) Dr. Alavi developed the OPTT TF-CBT program and will oversee the e-CBT therapy.<br><br>4) New combined approach using Ketamine and e-CBT for PTSD with chances for rapid and effective results.	1) Key informing is missing for the reviewers. Why is it relevant to conduct this research in Canada? Is it new? Are other researchers, including Canadian researchers employing ketamine in combination with psychotherapy treatments? The combined therapeutic approach should be put into context with other possible similar ongoing studies. It is also important to mention literature available for similar combined approach and to clearly state why and how this research is new and has better chances to succeed.

Overall Score (based on CIHR scale below)

Descriptor	Score	**Outcome**
Outstanding	4.5 – 4.9	**May Be Funded**
Excellent	4.0 – 4.4	
Very good	3.5 – 3.9	
Acceptable, but low priority	3.0 – 3.4	**May or May Not be Fundable**
Needs revision	2.5 – 2.9	
Needs major revision	2.0 – 2.4	
Seriously flawed	1.0 – 1.9	
Rejected	0.0-0.9	

Overall Adjudication Score: 4.5

Comments: Evidence from literature supports the hypothesis that the combination of a ketamine-based pharmacological approach with psychotherapeutic treatment may significantly improve symptoms in treatment resistant PTSD. This is a timing and relevant project that may lead to a much-needed rapid and effective new approach to treat PTSD. The ketamine project led by Dr. Vasques is leading to many new collaborations among Queen's researches and fostering new ideas. It has great chances to become a competitive CIHR proposal. Improvements in the text (as suggested) will help to make this a strong proposal.

Budget is appropriate. Great mix of young and established researchers with complimentary expertise.

Queen's University

Queen's University Department of Psychiatry Internal Research Grant Review

Title of Grant: Ketamine & eCBT for PTSD

Primary Investigator: Dr. Taras Reshtukha

Instructions:
Assess per the adjudication scale and list 3-5 strengths and/or weaknesses in point form in the space provided.

Adjudication Scale	
**4.5 – 4.9** Exceptional	**3.0-3.4** Very good however needs revision to be fundable
**4.0-4.4** Outstanding	**2.5-2.9** Needs major revision
**3.5-3.9** Excellent, may still require revision	**0.0-2.4** Seriously flawed

Originality/Scientific Merit: **Adjudication Score: 3.4**

Comments:

- Several different types of CBT exist for PTSD, including Prolonged Exposure (e.g., Foa) and Cognitive Processing Therapy (e.g., Monson) – should distinguish this in proposal regarding evidence base and re: nature of eCBT – more specifics of treatment. What is the role of exposure, trauma processing in TF-CBT? How will those be delivered asynchronously?
- *"Patients must have been receiving stable treatment for 2 months prior to enrolment in the study."* Meaning what? What is current standard treatment being offered? This needs clarification. Why two months?
- "*Patients with suicidal ideations and co-morbid substance use disorders (excluding opioids) will also be included in the study*." Why exclude opioids specifically but not other substances? Danger of ketamine + other substances?
- Any data on safety of asynchronous online psychotherapy for PTSD? Increased distress/suicidality? What to do if patient dissociates?
- Why no e-CBT stand alone & Ketamine stand alone groups? (4 study groups for comparison)? No treatment at all for controls in first phase? Or "stable treatment" of some sort? Without the 2 other comparison groups cannot speak to source of change in symptoms from combined treatment vs. no treatment.
- Sample size – assumption than n of 20 per group sufficient to see change - what is expected effect size of treatment?
- What about cost of ketamine? Not in budget
- More specifics re: hypotheses needed
- What about issues of access to technology? Rural area internet connection, access to devices, skills in using technology. Need to address in inclusion criteria and limitations.
-

Strengths:	Weaknesses:
1) Concept of medication potentiating/facilitating psychotherapy response is important (i.e., not just additive, but potentially interactive)<br><br>2) Both Ketamine & eTherapy growing areas of research interest<br><br>3)_Value of developing rapid treatments	1) Study design does not provide ability to identify source of treatment effects. Need additional treatment groups (Treatment A, Treatment B, Treatment A + B, Control)<br><br>2) Need to explain some methodological decisions (in comments above)<br><br>3) Study limitations & risks not addressed<br><br>4)_Need to fill in a few literature gaps (see comments)

Impact and Research Applicant(s)

**Adjudication Scale (Research impact and Research Applicant(s))**	
**4.5–4.9** Extremely significant impact appropriate team	**3.0-3.4** Moderate impact and/or poor team
**4.0-4.4** Very significant impact ,appropriate team	**2.5-2.9** Limited impact and/or poor team
**3.5-3.9** Significant impact, appropriate team	**0.0-2.4** Negligible impact and/or poor team

**Potential Impact and Team:**	**Adjudication Score:3.5**

Comments:

- Who is providing the asynchronous eCBT?
- Senior & junior investigators not clearly identified
- Plan for larger scale studies? Further grants?
- Cross division involvement? Divisions not clear
- Unclear how aligns with strategic framework – need more discussion of this
- Impact: If patients already coming in 2x/week for infusions, then what is value of eCBT from home vs in person? Proof of concept?
- Literature on combination of eCBT with medications (not just cbt + medications)? Literature on combo of ketamine with face-to-face CBT? Need to include this to speak to importance/novelty of study.

**Strengths:**	**Weaknesses:**
1) Adds to growing body of research on ketamine in treatment of mental disorders and specifically PTSD<br><br>2)Adds to body of research on eCBT, especially timely due to current limitations in face to face services<br><br>3) Adds to research on multi-modal & combined intervention approaches for PTSD	1) Value of online (i.e, from home) treatment component not clear if having to come in person for other aspects of treatment (i.e., why is eCBT + ketamine better than face to face CBT + ketamine?)<br><br>2 Need a plan for scale up/next steps<br><br>3) Need to more clearly articulate how this will grow collaboration & increase research capacity (one goal of these larger grants)

Overall Score (based on CIHR scale below)

Descriptor	Score	**Outcome**
Outstanding	4.5 – 4.9	**May Be Funded**
Excellent	4.0 – 4.4	
Very good	3.5 – 3.9	
Acceptable, but low priority	3.0 – 3.4	**May or May Not be Fundable**
Needs revision	2.5 – 2.9	
Needs major revision	2.0 – 2.4	
Seriously flawed	1.0 – 1.9	
Rejected	0.0-0.9	

Overall Adjudication Score:

Queen's UNIVERSITY

Comments:

This study has a number of strengths, as identified above. With some changes to the methodology it has the potential to provide very interesting and clinically relevant data on novel approaches to the treatment of PTSD. Before funding, however, the research team should address the areas outlined above. The critical change is to add two study groups to the design (and consider sample size) to allow for meaningful interpretation of the findings. Specifically, instead of just Treatment A + Treatment B vs. Control, the 2 groups Treatment A and Treatment B should be added for cross comparison. I would also consider exploring the value of eCBT vs in person CBT in combination with ketamine (although that might be a separate study.)
