# Supplementary material for: Combining Ketamine and Internet-Based Cognitive Behavioral Therapy for the Treatment of Posttraumatic Stress Disorder: Protocol for a Randomized Controlled Trial
Source: JMIR Res Protoc. 2021 Jul 20;10(7):e30334. doi: 10.2196/30334 (PMC8335614; doi:10.2196/30334)
Supplement: Multimedia Appendix 4 [file resprot_v10i7e30334_app4.pdf]

August 31, 2020

Re: Department of Psychiatry Internal Grant Competition 2020.

Dear Drs Alavi and Reshetukha ,

There were many excellent applications to the Department of Psychiatry Internal Grant Competition this year and as a result several deserving proposals remain unfunded.

However, I am pleased to inform you that your application entitled "Ketamine & eCBT for PTSD" has been awarded $17,000.

Please see the attached reviewer comments for feedback.

Please communicate the results of this competition to your co-investigators, and provide Jody Burns (burnsj@providencecare.ca) with the account number into which these funds should be transferred.

Congratulations!

Sincerely,

Dr. Dianne Groll
Chair, Department of Psychiatry Research Committee
