# Supplementary material for: Combining Ketamine and Internet-Based Cognitive Behavioral Therapy for the Treatment of Posttraumatic Stress Disorder: Protocol for a Randomized Controlled Trial
Source: JMIR Res Protoc. 2021 Jul 20;10(7):e30334. doi: 10.2196/30334 (PMC8335614; doi:10.2196/30334)
Supplement: Multimedia Appendix 5 [file resprot_v10i7e30334_app5.pdf]

CONSORT-EHEALTH Checklist V1.6.2 Report	Manuscript Number	30334
**(based on CONSORT-EHEALTH V1.6), available at [http://tinyurl.com/consort-ehealth-v1-6].**		
**Date completed**		
5/31/2021 5:30:37		
**by**		
Aaron Philipp-Muller		
Combining Ketamine and Online Cognitive Behavioural Therapy for the Treatment of Post Traumatic Stress Disorder: Protocol for a Randomized Waitlist-Control Trial		
TITLE		
**1a-i) Identify the mode of delivery in the title**		
"Combining Ketamine and Internet-Based Cognitive Behavioural Therapy for the Treatment of Post Traumatic Stress Disorder: Protocol for a Randomized Control Trial"		
**1a-ii) Non-web-based components or important co-interventions in title**		
**1a-iii) Primary condition or target group in the title**		
"Combining Ketamine and Internet-Based Cognitive Behavioural Therapy for the Treatment of Post Traumatic Stress Disorder: Protocol for a Randomized Control Trial"		
ABSTRACT		
**1b-i) Key features/functionalities/components of the intervention and comparator in the METHODS section of the ABSTRACT**		
"20 participants with refractory PTSD will be randomly assigned either to an experimental group receiving a combination of ketamine and therapist-administered e-CBT over 14 weeks, or they will be assigned to a waitlist-control group at the end of which they will receive the experimental treatment. Both groups will be assessed for symptoms of PTSD and comorbid disorders before treatment, at the halfway point, and at the end of the experiment."		
**1b-ii) Level of human involvement in the METHODS section of the ABSTRACT**		
"20 participants with refractory PTSD will be randomly assigned either to an experimental group receiving a combination of ketamine and therapist-administered e-CBT over 14 weeks, or they will be assigned to a waitlist-control group at the end of which they will receive the experimental treatment. Both groups will be assessed for symptoms of PTSD and comorbid disorders before treatment, at the halfway point, and at the end of the experiment."		
**1b-iii) Open vs. closed, web-based (self-assessment) vs. face-to-face assessments in the METHODS section of the ABSTRACT**		
"recruited from a community clinic"		
**1b-iv) RESULTS section in abstract must contain use data**		
This information is not yet available as the experiment has not yet been conducted.		
**1b-v) CONCLUSIONS/DISCUSSION in abstract for negative trials**		
This information is not yet available as the study has yet to be conducted.		
INTRODUCTION		
**2a-i) Problem and the type of system/solution**		
"Post-Traumatic Stress Disorder (PTSD) is a chronic and debilitating mental illness occurring in 3.5% of North American adults with a lifetime prevalence of 8%, or approximately 28 million people [1]. PTSD develops after direct or indirect exposure to a psychologically traumatic incident, leading to a host of cog-nitive, emotional and behavioural symptoms [1]. While most patients recover after a psychological trau-ma, approximately 20% of individuals remain chronically symptomatic or experience delayed onset of PTSD [2]. PTSD is a particularly refractory disorder, lasting for years, with many patients still displaying symptoms 20 years after a trauma [3]. By some accounts, PTSD prevalence may grow as time increases after a trauma [4]. PTSD is also associated with high comorbidity rates with depression, anxiety and sub-stance abuse disorder. [5,6]. Patients with PTSD are also 4 times more likely to attempt suicide than trauma survivors without PTSD [7]. Taken together, these features draw attention to the urgent need of effective strategies for treating this disorder.<br><br>A number of empirically supported psychotherapeutic treatments are available for PTSD, with Trauma-Focused Cognitive Behavioural Therapy and Eye Movement Desensitization and Reprocessing Therapy (EMDR) being among the most effective [8, 9]. However, traditional treatments have a considerable non-response rate [10, 11]. A number of pharmacotherapies have also become available to treat PTSD, with SSRIs such as Paroxetine and Sertraline seeing the greatest success [12]. Unfortunately, pharmacothera-pies also have a large non-response rate [12]. As a result there remains a sizeable proportion of patients with PTSD that are treatment resistant. The goal of the current study will be to provide a greater reduction in symptoms compared to current available therapeutic options for treatment resistant patients."		
**2a-ii) Scientific background, rationale: What is known about the (type of) system**		
"Ketamine is a promising research avenue for treating refractory PTSD. It is primarily a glutamate antago-nist at the N-methyl-D-aspartate (NMDA) receptor, and it has had considerable success in rapidly reduc-ing symptoms of mood disorders [13]. Ketamine is thought to function by disengaging an established pat-tern of thought [14,15], which in the case of PTSD would involve counteracting impaired fear extinction as ketamine increases neuroplasticity towards fear learning [16,17] . It is important to note that its exact mechanism of action in the treatment of emotional disorders is only partly understood. In terms of the de-velopment of PTSD, recent advances have generally implicated the NMDA receptor, where rodents sub-jected to chronic stress have elevated gene-expression for production of NMDA receptors in the ventral hippocampus in comparison to control subjects [18]. Moreover, in humans, the prefrontal cortex and amygdala are connected by glutamatergic projections, suggesting that glutamate mediates a fear response [19]. Ketamine, in turn, has had considerable success in treating refractory PTSD, where it significantly reduces symptoms in comparison to an active placebo for treatment-resistant patients [20].<br><br>While ketamine opens treatment options to a new patient cohort, a purely pharmacological approach would be an oversimplification given the nature of PTSD, as the disorder develops in the wake of a trau-ma and cannot develop from pathological neurochemistry or neuroanatomy alone. Moreover, ketamine's effects wear off in under a week [20, 21], and repeated infusions can have potentially negative outcomes on cognitive and physical health in the long-term [22]. One potential way to prolong its effects is to capi-talize on ketamine's role in facilitating fear extinction by combining it with psychotherapy. This is partly due to psychotherapeutic interventions having more longevity than pharmacological techniques for reduc-ing symptoms of PTSD [23]. At the moment, there have been very few studies combining ketamine and psychotherapy to treat PTSD. As of May 2021, there were 4 studies listed on the ClinicalTrials.gov data-base investigating ketamine used in combination with psychotherapy to treat PTSD (NCT02727998; NCT02766192; NCT04560660; NCT03960658). The results of these studies have been promising, where patients given a combination of ketamine and Prolonged Exposure therapy or Mindfulness-based Extinction and Reconsolidation therapy have a greatly prolonged therapeutic response in comparison to ketamine alone [24,25]. The proposed study would therefore attempt to build on these findings by exam-ining other types of psychotherapy in conjunction with ketamine to treat PTSD."		
**Does your paper address CONSORT subitem 2b?**		
"In this study, we will focus on Trauma-focused Cognitive-Behavioural Therapy (TF-CBT). As men-tioned above, TF-CBT is a well-established form of psychotherapy and is considered among the most effective forms of psychotherapy for PTSD immediately post treatment and at follow-up [8, 26, 27]. However, as mentioned above, there is still a considerable proportion of PTSD patients that do not re-spond to TF-CBT. One challenge associated with CBT is in optimizing inhibitory learning, which is in-herently challenging for patients with PTSD [28,29]. Ketamine treatment may address this challenge as it acts to boost neuroplasticity. Another challenge with CBT is its resource intensive nature, with large as-sociated costs and waiting times [30, 31]. One variant of CBT that addresses this second challenge is in-ternet-delivered (i.e. online) Cognitive Behavioural Therapy (e-CBT), which has equivalent efficacy as face-to-face CBT for treating PTSD as seen in several meta-analyses [32, 33]. Unfortunately, there has yet to be a study examining the interaction of ketamine treatment with e-CBT or CBT in general. There-fore, this study will assess whether ketamine combined with e-CBT significantly reduces symptoms of PTSD in treatment-resistant patients. We hypothesize that ketamine treatment alongside e-CBT will re-duce symptoms of treatment resistant PTSD significantly more than a waitlist control, thereby providing preliminary evidence that these two treatments can be successfully combined."		
METHODS		
**3a) CONSORT: Description of trial design (such as parallel, factorial) including allocation ratio**		
"This study has a randomized, open-label parallel design." "recruiting 10 participants per treatment group"		
**3b) CONSORT: Important changes to methods after trial commencement (such as eligibility criteria), with reasons**		
This information is not yet available as the study has yet to be conducted.		
**3b-i) Bug fixes, Downtimes, Content Changes**		
This information is not yet available as the study has yet to be conducted.		
**4a) CONSORT: Eligibility criteria for participants**		

"Inclusion Criteria:<br>i) Provide oral consent;<br>ii) Patients will be 18-65 years of age at the start of the study;<br>iii) Patients will be diagnosed with PTSD by a psychiatrist on the team as outlined in the DSM-5 to quali-fy at least as moderate presentation on the Clinician Administered PTSD Scale (CAPS-5) with a score of at least 50;<br>iv) Patients will be treatment resistant, where they will have received at least 2 different types of prior treatment, including any combination of Selective Serotonin Reuptake Inhibitors, Serotonin Norepineph-rine Reuptake Inhibitors or Trauma-Focused Cognitive-Behavioural Therapy, and all prior treatments will have produced less than a 50% reduction in the participant's symptoms;<br>v) Patients will be on stable treatment for at least 8 weeks prior to screening, with no alterations to treat-ment regimen in order to isolate for treatment effects;<br>vi) If a participant is female and of childbearing potential, they must be using an effective method of con-traception as ketamine can be harmful to the neural development of an embryo/fetus;<br>vii) Participants must be able to speak and read in English, and have consistent and reliable access to the internet in order to complete the e-CBT course;<br>viii) Participants must agree to adhere to the study protocol.<br>Exclusion Criteria:<br>i) Prior hypersensitivity/allergy to ketamine.<br>ii) Hypomanic/manic episodes, bipolar disorder, acute psychosis, and/or schizophrenia;<br>iii) Opioid use disorder, current use of opioids, or treatment with Naltrexone;<br>iv) Currently pregnant, postpartum, or breastfeeding;<br>v) Untreated or inadequately controlled hypertension and/or cardiovascular disease;<br>vi) Elevated intracranial pressure;<br>vii) Renal or hepatic disease;<br>viii) Antisocial personality disorder and/or active homicidal ideation."		
**4a-i) Computer / Internet literacy**		
Inclusion criteria include: "vii) Participants must be able to speak and read in English, and have consistent and reliable access to the internet in order to complete the e-CBT course"		
**4a-ii) Open vs. closed, web-based vs. face-to-face assessments:**		
"The referring physicians will first be given flyers containing a brief description of the study with inclusion/exclusion criteria and contact information. They will then provide incoming patients that match the basic criteria with a copy of the flyer, informing them of this study. Patients that are interested in participating will contact the study coordinator, who will call the patient to conduct a prescreening interview, asking basic questions to determine if the patient may be eligible. If the patient passes this interview, the patient will proceed to the screening phase." The screening sessions will be face to face over an online interface, and the ketamine screening sessions and appointments will be in-person. The psychotherapy will asynchronous and online.		
**4a-iii) Information giving during recruitment**		
This project included an information meeting after the 3rd screening visit where participants are briefed on all the procedures that will take place as part of the project and all risks and benefits where they will have the opportunity to provide consent. All of these features are required by the research ethics board. "This protocol has been approved by the Queen's University Health Sciences and Affiliated Teaching Hospitals Research Ethics Board"		
**4b) CONSORT: Settings and locations where the data were collected**		
"...enrolled in the study based on referrals from outpatient psychiatry at Hotel Dieu Hospital in Kingston, Ontario, Canada" "Patients in the combination therapy group will also receive ketamine infusions at the Providence Care Hospital Ketamine Clinic"		
**4b-i) Report if outcomes were (self-)assessed through online questionnaires**		
The following online questionnaires will be completed by participants:<br><br>"iv) Sheehan Disabilities Scale (SDS) to provide insight into a patient's social and occupational function-ing [44];<br>v) Global Assessment of Functioning Scale (GAF) to provide additional insight into social/occupational function [45]."		
**4b-ii) Report how institutional affiliations are displayed**		
On the recruitment flyer and consent form, participants are informed that this study is occurring through Queen's University and KHSC, and the related insignia are present as a header on all material given to participants.		
**5) CONSORT: Describe the interventions for each group with sufficient details to allow replication, including how and when they were actually administered**		
**5-i) Mention names, credential, affiliations of the developers, sponsors, and owners**		
"Acknowledgments<br>This study was funded by the Queen's Faculty of Psychiatry Internal Faculty Grant ($17,000). The fund-ing source had no role in the study design, study execution, data collection, data analysis, or data interpre-tation.<br>Conflicts of Interest<br>Dr. Nazanin Alavi, M.D. is an assistant professor of psychiatry at Queen's University and has co-founded the care delivery platform in use (OPTT) and has ownership stakes in OPTT Inc."		
**5-ii) Describe the history/development process**		
"The format for the content of the modules and the overall structure of the therapy course and delivery plat-form is based on previous work by Alavi et al on e-CBT for other mood and anxiety disorders [37, 38, 39]."		
**5-iii) Revisions and updating**		
This information is not available as the experiment has not yet been conducted.		
**5-iv) Quality assurance methods**		
This question is not applicable in the case of this study.		
**5-v) Ensure replicability by publishing the source code, and/or providing screenshots/screen-capture video, and/or providing flowcharts of the algorithms used**		
**5-vi) Digital preservation**		
**5-vii) Access**		
"All online sessions will take place through the Online PsychoTherapy Tool (OPTT), a secure, cloud-based online service for hosting asynchronous psychotherapy. Patients will first be introduced to their therapist, who will then email the patient a link to their weekly module"		
**5-viii) Mode of delivery, features/functionalities/components of the intervention and comparator, and the theoretical framework**		
"Participants from the combination therapy group will begin an e-CBT program, which will involve a 14-week course of Trauma-Focused Cognitive Behavioural Therapy (TF-CBT). The content of the therapy course will mirror the in-person Trauma-Focused Cognitive Behavioural Therapy and Cognitive Pro-cessing Therapy intervention for PTSD [36]. The format for the content of the modules and the overall structure of the therapy course and delivery platform is based on previous work by Alavi et al on e-CBT for other mood and anxiety disorders [37, 38, 39]. All online sessions will take place through the Online PsychoTherapy Tool (OPTT), a secure, cloud-based online service for hosting asynchronous psychother-apy. Patients will first be introduced to their therapist, who will then email the patient a link to their week-ly module, which will be presented to the patient in the form of approximately 30 presentation slides. Each week's module will highlight a particular topic, and will include general information, an overview of skills, and homework to be completed at any time within the week. OPTT will save a patient's progress so that they may work at their own pace, resuming when it is convenient for them. This homework will take approximately 40 minutes in total to complete and will be submitted within one week through OPTT to the therapist who will provide personalized feedback across the same platform. Because of the prede-signed format of the modules, on average, developing this feedback takes a therapist approximately 15-20 minutes per patient. Thus, the time needed to respond to each patient is reduced, increasing the number of patients each therapist can handle.<br>Therapist feedback will involve content that seeks to build rapport, review skills, review the con-tent of the patient's homework, and provide constructive feedback. A more detailed explanation on this structure can be found in Online Cognitive Behavioral Therapy: An e-Mental Health Approach to De-pression and Anxiety, a book by Alavi and Omrani [40]. Although the homework and clinician feedback are considered the main means of communication between therapists and participants, participants can al-so communicate with their therapist through a secure chat function that is found directly within the OPTT. This is mainly used to let participants ask further questions about their care, if anything is not clear. Any technical issues will be handled through the OPTT technical support team, which participants will have access to at all times during the program. The patient care team (i.e. the therapist and the psychiatrist) will also be able to securely communicate through the OPTT to make decisions regarding each patient's care path.<br>Online Module Content: The TF-CBT is focused on strategies that would be helpful in handling stress and mood problems related to the traumas that patients have experienced in their life. The program helps the patients to independently manage their emotions, thoughts and behaviours. In this course, trauma-informed care will be discussed. The course is designed specifically to address the need for healing from traumatic events and to facilitate recovery through therapy. Topics will include stuck points, identifying events, index events, problematic thinking, challenging beliefs, as well as safety and trust.<br>Training: All therapists are research assistants hired by the co-principal investigator leading the online psychotherapy portion of the research. They will undergo training in psychotherapy and additional train-ing from a psychiatrist on the research team before any interaction with participants. During this training, therapists complete feedback on practice homework, which is reviewed by a psychiatrist on the research team to ensure adequate quality of work. All therapists are supervised by the lead psychiatrist, who is an expert in the area of electronically delivered psychotherapy [40]. Feedback is always reviewed by the lead psychiatrist, before submission to the participants."		
**5-ix) Describe use parameters**		

**5-x) Clarify the level of human involvement**		
**5-xi) Report any prompts/reminders used**		
"Finally, if a patient does not complete their homework for the week, they will receive three weekly emails, after which they will be removed from the experiment."		
**5-xii) Describe any co-interventions (incl. training/support)**		
No co-interventions are planned.		
**6a) CONSORT: Completely defined pre-specified primary and secondary outcome measures, including how and when they were assessed**		
"Outcome Evaluation<br>Patient outcome will be measured through clinical interviews and questionnaires, completed at the start (baseline measurement) and end of treatment. The primary measure (see next paragraph) will also have midway assessments at 4 weeks and 8 weeks through treatment. All questionnaire data will be adminis-tered electronically to the patients through OPTT alongside their e-CBT session that week. Interviews and observational data will be collected either in person or through virtual (video) appointments.<br>Primary Outcome<br>The primary outcome measure will be the CAPS-5 interview [34]. Treatment response will be defined as a 50% reduction in the participant's scores at the end of the 14-week period in comparison to the partici-pant's scores at baseline. Non-response will be defined as less than a 50% reduction in scores. Remission will be defined as a 75% reduction in scores while relapse will be defined as a temporary treatment re-sponse or remission at one or both of the halfway points with a return to non-response at the final as-sessment.<br>Secondary Outcome<br>Secondary outcome measures will include the following:<br>i) Montgomery Asberg Depression Rating Scale (MADRS) to measure symptoms of depression [41];<br>ii) Columbia-Suicide Severity Rating Scale, risk assessment version (C-SSRS) to measure suicidality [42];<br>iii) Clinical Global Impression (CGI) to measure a patient's overall clinical presentation from a clinician's perspective and to provide inter-rater reliability [43];<br>iv) Sheehan Disabilities Scale (SDS) to provide insight into a patient's social and occupational function-ing [44];<br>v) Global Assessment of Functioning Scale (GAF) to provide additional insight into social/occupational function [45]."		
**6a-i) Online questionnaires: describe if they were validated for online use and apply CHERRIES items to describe how the questionnaires were designed/deployed**		
**6a-ii) Describe whether and how "use" (including intensity of use/dosage) was defined/measured/monitored**		
**6a-iii) Describe whether, how, and when qualitative feedback from participants was obtained**		
**6b) CONSORT: Any changes to trial outcomes after the trial commenced, with reasons**		
"...enrolled in the study based on referrals from outpatient psychiatry at Hotel Dieu Hospital in Kingston, Ontario, Canada" "Patients in the combination therapy group will also receive ketamine infusions at the Providence Care Hospital Ketamine Clinic"		
**7a) CONSORT: How sample size was determined**		
**7a-i) Describe whether and how expected attrition was taken into account when calculating the sample size**		
**7b) CONSORT: When applicable, explanation of any interim analyses and stopping guidelines**		
"Outcome Evaluation<br>Patient outcome will be measured through clinical interviews and questionnaires, completed at the start (baseline measurement) and end of treatment. The primary measure (see next paragraph) will also have midway assessments at 4 weeks and 8 weeks through treatment. All questionnaire data will be adminis-tered electronically to the patients through OPTT alongside their e-CBT session that week. Interviews and observational data will be collected either in person or through virtual (video) appointments.<br>Primary Outcome<br>The primary outcome measure will be the CAPS-5 interview [34]. Treatment response will be defined as a 50% reduction in the participant's scores at the end of the 14-week period in comparison to the partici-pant's scores at baseline. Non-response will be defined as less than a 50% reduction in scores. Remission will be defined as a 75% reduction in scores while relapse will be defined as a temporary treatment re-sponse or remission at one or both of the halfway points with a return to non-response at the final as-sessment.<br>Secondary Outcome<br>Secondary outcome measures will include the following:<br>i) Montgomery Asberg Depression Rating Scale (MADRS) to measure symptoms of depression [41];<br>ii) Columbia-Suicide Severity Rating Scale, risk assessment version (C-SSRS) to measure suicidality [42];<br>iii) Clinical Global Impression (CGI) to measure a patient's overall clinical presentation from a clinician's perspective and to provide inter-rater reliability [43];<br>iv) Sheehan Disabilities Scale (SDS) to provide insight into a patient's social and occupational function-ing [44];<br>v) Global Assessment of Functioning Scale (GAF) to provide additional insight into social/occupational function [45]."		
**8a) CONSORT: Method used to generate the random allocation sequence**		
"Participants will be entered into the study through computer-generated block randomization by the study coordinator, recruited in pairs so that 10 patients will receive the experimental condition and 10 will be in the control group"		
**8b) CONSORT: Type of randomisation; details of any restriction (such as blocking and block size)**		
"Participants will be entered into the study through computer-generated block randomization by the study coordinator, recruited in pairs so that 10 patients will receive the experimental condition and 10 will be in the control group"		
**9) CONSORT: Mechanism used to implement the random allocation sequence (such as sequentially numbered containers), describing any steps taken to conceal the sequence until interventions were assigned**		
"Participants will be entered into the study through computer-generated block randomization by the study coordinator, recruited in pairs so that 10 patients will receive the experimental condition and 10 will be in the control group"		
**10) CONSORT: Who generated the random allocation sequence, who enrolled participants, and who assigned participants to interventions**		
"Participants will be entered into the study through computer-generated block randomization by the study coordinator, recruited in pairs so that 10 patients will receive the experimental condition and 10 will be in the control group"		
**11a) CONSORT: Blinding - If done, who was blinded after assignment to interventions (for example, participants, care providers, those assessing outcomes) and how**		
**11a-i) Specify who was blinded, and who wasn't**		
No blinding will take place in this study.		
**11a-ii) Discuss e.g., whether participants knew which intervention was the "intervention of interest" and which one was the "comparator"**		
**11b) CONSORT: If relevant, description of the similarity of interventions**		
This question is not applicable in the case of this study.		
**12a) CONSORT: Statistical methods used to compare groups for primary and secondary outcomes**		
"If the assumptions are met, a 2 x 4 mixed effects Analysis of Variance (ANOVA; P=.05) will be con-ducted to determine the main effects of the 2 factors, 'time' and 'condition', as well as the interaction ef-fect between time and condition on PTSD symptom outcome for CAPS-5 symptom severity. 'Time' con-sists of 4 levels (before treatment, 4 weeks through treatment, 8 weeks through treatment, and end of treatment), while 'condition' includes 2 levels (experimental condition and waitlist control condition). Simple main effects will be tested for time and condition, and a Bonferroni p-value adjustment will be made for the 'time' factor. Post hoc tests will be conducted on the 'time' variable with a Tukey's range test.<br><br>The secondary outcome measures are exploratory in nature, and will be conducted with a 2x2 mixed effects ANOVA (P=.05), where 'time' has only 2 levels and no post-hoc tests will be conducted. All other components will remain the same."		
**12a-i) Imputation techniques to deal with attrition / missing values**		
There will not be missing data as participants entire file will be deleted if they do not finish the experiment.		
**12b) CONSORT: Methods for additional analyses, such as subgroup analyses and adjusted analyses**		
There are no additional analyses planned at this point.		
RESULTS		
**13a) CONSORT: For each group, the numbers of participants who were randomly assigned, received intended treatment, and were analysed for the primary outcome**		
This information is not yet available as this study has not yet been conducted.		
**13b) CONSORT: For each group, losses and exclusions after randomisation, together with reasons**		
This information is not yet available as this study has not yet been conducted.		
**13b-i) Attrition diagram**		

**14a) CONSORT: Dates defining the periods of recruitment and follow-up**		
Note that this information is anticipatory: "Recruitment of participants is set to begin in the end of May 2021, based on clinician referrals from Out-patient Psychiatry at Hotel Dieu Hospital. Recruitment will take place with approximately 2 participants added each month until February 2022."		
**14a-i) Indicate if critical "secular events" fell into the study period**		
**14b) CONSORT: Why the trial ended or was stopped (early)**		
This information is not yet available as this study has not yet been conducted.		
**15) CONSORT: A table showing baseline demographic and clinical characteristics for each group**		
This information is not yet available as the study has not yet been conducted.		
**15-i) Report demographics associated with digital divide issues**		
This information is not yet available as the study has not yet been conducted.		
**16a) CONSORT: For each group, number of participants (denominator) included in each analysis and whether the analysis was by original assigned groups**		
**16-i) Report multiple "denominators" and provide definitions**		
This information is not yet available as the study has not yet been conducted.		
**16-ii) Primary analysis should be intent-to-treat**		
**17a) CONSORT: For each primary and secondary outcome, results for each group, and the estimated effect size and its precision (such as 95% confidence interval)**		
This information is not yet available as the study has not yet been conducted.		
**17a-i) Presentation of process outcomes such as metrics of use and intensity of use**		
**17b) CONSORT: For binary outcomes, presentation of both absolute and relative effect sizes is recommended**		
This information is not yet available as the study has not yet been conducted.		
**18) CONSORT: Results of any other analyses performed, including subgroup analyses and adjusted analyses, distinguishing pre-specified from exploratory**		
No other analyses are planned for this study.		
**18-i) Subgroup analysis of comparing only users**		
**19) CONSORT: All important harms or unintended effects in each group**		
This information is not yet available as the study has not yet been conducted.		
**19-i) Include privacy breaches, technical problems**		
**19-ii) Include qualitative feedback from participants or observations from staff/researchers**		
DISCUSSION		
**20) CONSORT: Trial limitations, addressing sources of potential bias, imprecision, multiplicity of analyses**		
**20-i) Typical limitations in ehealth trials**		
"First, if the treatment successfully reduces symptoms, the current protocol offers no way to determine the source of the improvement. Symptom improvement could be attributed primarily to the e-CBT, or to the ketamine, or both equally. A second issue is that this study does not offer insight into the post-treatment timeline for relapse, as the last assessment is immediately following treatment. Another issue is due to the unblinded nature of the study, participants may be biased as a result. Finally, the current out-come measures do not shed light on the mechanism by which either of the experimental treatments influences the other."		
**21) CONSORT: Generalisability (external validity, applicability) of the trial findings**		
**21-i) Generalizability to other populations**		
**21-ii) Discuss if there were elements in the RCT that would be different in a routine application setting**		
**22) CONSORT: Interpretation consistent with results, balancing benefits and harms, and considering other relevant evidence**		
**22-i) Restate study questions and summarize the answers suggested by the data, starting with primary outcomes and process outcomes (use)**		
"The main anticipated findings of this study will evaluate the efficacy of a novel intervention in a treatment-resistant patient population."		
**22-ii) Highlight unanswered new questions, suggest future research**		
Other information		
**23) CONSORT: Registration number and name of trial registry**		
"ClinicalTrials.gov database - NCT04771767"		
**24) CONSORT: Where the full trial protocol can be accessed, if available**		
See multimedia appendix 6.		
**25) CONSORT: Sources of funding and other support (such as supply of drugs), role of funders**		
"This study was funded by the Queen's Faculty of Psychiatry Internal Faculty Grant ($17,000). The funding source had no role in the study design, study execution, data collection, data analysis, or data interpretation."		
**X26-i) Comment on ethics committee approval**		
**x26-ii) Outline informed consent procedures**		
**X26-iii) Safety and security procedures**		
This information is explored in depth in multimedia appendix 2.		
**X27-i) State the relation of the study team towards the system being evaluated**		
"Dr. Nazanin Alavi, M.D. is an assistant professor of psychiatry at Queen's University and has co-founded the care delivery platform in use (OPTT) and has ownership stakes in OPTT Inc."		
